# The association between dietary folate intake and risk of colorectal cancer incidence: A systematic review and dose‒response meta-analysis of cohort studies

**DOI:** 10.1016/j.heliyon.2024.e33564

**Published:** 2024-06-26

**Authors:** Masoumeh Khalighi Sikaroudi, Sepideh Soltani, Roya Kolahdouz-Mohammadi, Roya Imanifard, Shima Abdollahi, Hossein Shahinfar, Gholamreza Mohammadi Farsani

**Affiliations:** a Department of Clinical Nutrition, Tehran University of Medical Sciences, Tehran, Iran; b Yazd Cardiovascular Research Center, Noncommunicable Diseases Research Institute, Shahid Sadoughi University of Medical Sciences, Yazd, Iran; c Department of Nutrition, School of Public Health, Iran University of Medical Sciences, Tehran, Iran; d Department of Nutrition, School of Health, North Khorasan University of Medical Sciences, Bojnurd, Iran

**Keywords:** Colorectal cancer, Dietary folate, Cohort study, Meta-analysis, Dose response

## Abstract

**Background:**

Dietary components can influence the incidence of colorectal cancer (CRC). Folate is one of the compounds that plays an essential role in the formation of DNA structures, which can lead to or prevent tumorigenesis. The present study is the first systematic review and dose–response meta-analysis of cohort studies evaluating the association between dietary folate intake and the risk of CRC.

**Methods:**

The PubMed/Medline, Scopus, and ISI Web of Science databases were systematically searched for cohort studies that assessed the association between folate intake and CRC up to January 2024. Summary relative risks (RRs) and 95 % confidence intervals (CIs) were calculated using a random effects model. Also, linear and nonlinear dose-response analyses were conducted for the dose-response associations between folate intake and risk of CRC.

**Results:**

Eighteen prospective cohort studies with 931,469 participants, 14,860 CRC patients, 3536 colon cancer (CC) patients, and 1075 rectal cancer (RC) patients were included in the analysis. The summary RR of CRC for each 100-μg increase in dietary folate intake was 0.97 (95 % CI: 0.95–0.99, I^2^: 0.0 %, P-heterogeneity: 0.616), which can be related to BMI (0.97 (95 % CI: 0.95–0.99)); a more protective effect was also observed in subjects who drank alcohol (0.97 (95 % CI: 0.95–0.99)) and those who smoked (0.97 (95 % CI: 0.95–0.99)). Additionally, it was positively related to a 7 % lower risk of CC (0.93 (95 % CI: 0.87–0.99, I^2^: 33.7 %, P-heterogeneity: 0.159)), and the null relation for RC was 0.98 (95 % CI: 0.90–1.08), I^2^: 16.6 %, P-heterogeneity: 0.309). There was evidence of nonlinearity in which up to 500 μg/day dietary folate intake was inversely associated with CC (P nonlinearity = 0.04).

**Conclusion:**

The findings showed an inverse association between dietary folate intake and the risk of CRC, especially in high-risk persons, those who have a higher BMI, alcohol drinkers, and smokers.

## Highlights

• Folate has a critical role in DNA synthesis.
• Folate deficiency in cells leads to DNA structure disturbance and can induce tumorigenesis.
• A systematic review and dose response meta-analysis of 18 prospective cohort studies with 931,469 participants.
• 14,860 colorectal cancer cases, 3536 cases of colon cancer, and 1075 cases of rectal cancer.
• Inverse association between each 100-μg increase in dietary folate intake and risk of colorectal and colon cancer.

## Graphical abstract

**Figure:**
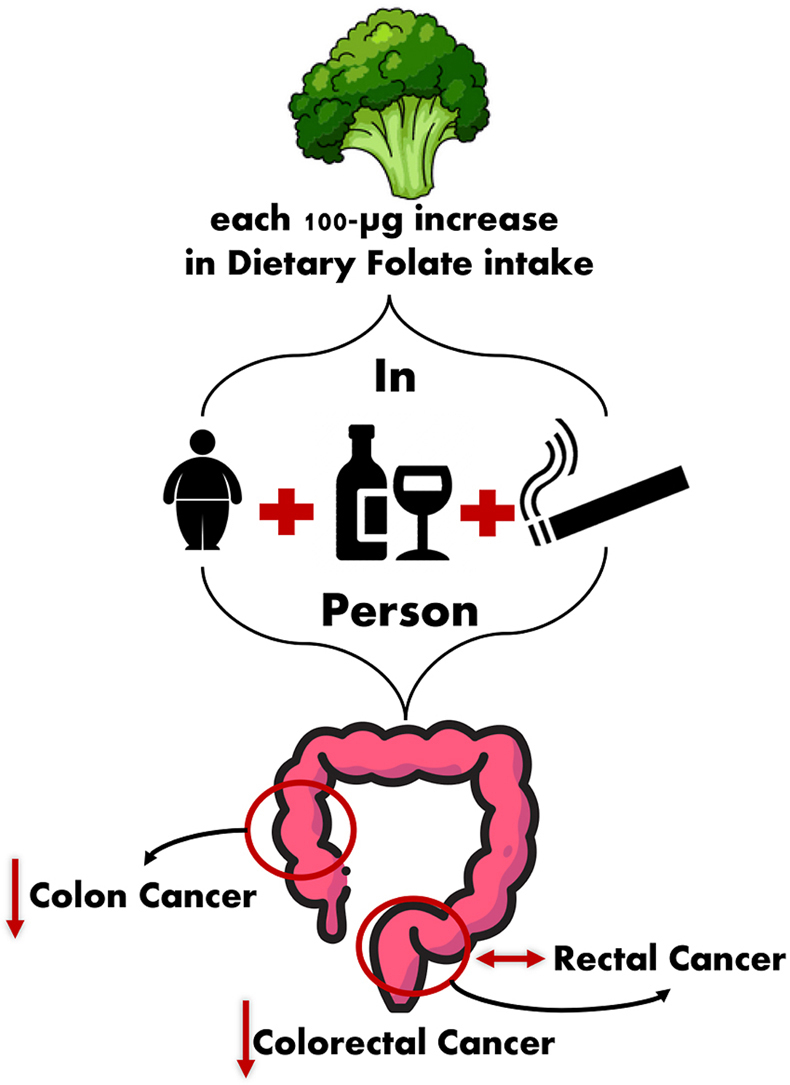

## 1 Introduction

Colorectal cancer (CRC) is known as the third most common malignancy and the second cause of cancer mortality [1,2]. A recent study reported that “*There are estimated 1.93 million new CRC cases diagnosed, and 0.94 million CRC caused deaths in 2020 worldwide, representing 10 % of the global cancer incidence and 9.4 % of all cancer caused deaths*” [2]. Several risk factors, such as age, sex, genetics, and largely modifiable lifestyle patterns, including smoking, alcohol consumption, low physical activity, not a suitable diet, and obesity, may affect the incidence of CRC [[3], [4], [5], [6], [7]]. Due to the increasing global incidence of CRC, the most important strategy is prevention, the main solution for which is changing lifestyles. Lower intake of fruits and vegetables and higher red meat intake are improper eating habits related to an increased risk of CRC [8].

Folate is one of the essential water-soluble B vitamin groups that is abundant in plant-based foods such as grains, legumes, and green leafy vegetables. The recommended dietary allowance (RDA) of folate for adults is 400 μg [9]. Since folate is a water-soluble vitamin and does not have enough storage in the body, if there is not enough folate in the diet, the level of this vitamin decreases at the cellular level [10]. The folate's key role in the cell is through methylation pathways during DNA synthesis [11]. Impaired DNA methylation due to folate deficiency causes DNA structure disruption and cell division. Due to this role, excessive folate intake has been studied extensively as a probable component of CRC incidence and development [[12], [13], [14]]. On the other hand, folic acid, which is found in fortified foods and supplements, might lead to changes in epigenetic structures as a source of methyl group provider. Some studies have hypothesized that excess folate intake from RDA recommended by dietary fortification or supplementation can cause concerns about abnormal cell division and cancer development [15,16]. However, the results of these studies are conflicting, and to confirm these findings additional studies are needed.

Recent trials or observational publications have been conducted to determine the effect of folate intake on the risk of CRC. These results are controversial, but most of studies reported an reverse association between folate intake and CRC incidence [[17], [18], [19], [20]]. Additionally, previous systematic and meta-analyses of observational (case‒control plus cohort) and clinical trials [[21], [22], [23], [24], [25], [26]] have shown a protective or no effect on CRC risk. These studies showed that the relative risk of CRC incidence was less in individuals with higher dietary folate intake than in those with lower one [21,23]. However, no meta-analysis has been specifically designed on prospective cohorts, and the current systematic review and meta-analysis as a first time aimed to assess the effect of different dosages of dietary folate intake on the risk of colorectal cancer incidence in prospective cohort studies that have not been reviewed previously.

## 2 Method

A systematic review and meta-analysis were conducted and reported according to the Meta-analysis of Observational Studies in Epidemiology (MOOSE) checklist [27]. The study protocol was registered in the PROSPERO International Prospective Register of Systematic Reviews (CRD42022316493).

### 2.1 Search strategy

A systematic literature search was performed in the PubMed/Medline, Scopus, and ISI Web of Science online databases before January 2024 without any language restriction. The search terms used were “folate” OR “dietary folate” AND “colorectal cancer” OR “colon cancer” OR “rectal cancer” AND “cohort”, and the full database search strategy is available in Supplementary Table 1.

### 2.2 Eligibility and study selection

The titles, abstracts, and full texts of the articles were reviewed by three reviewers (S.S., M.K.S., and RKM). The inclusion criteria for relevant articles were as follows: 1) published prospective cohort, nested case, or control–case cohort studies; 2) were conducted among adults (older than 18 years); 3) reported dietary folate intake as an exposure and at least in two categories; 4) reported incidence of colorectal, colon, or rectal cancer as a study outcome; 5) reported risk estimates (relative risk (RR) or hazard ratio (HR) or odds ratio (OR)) and their corresponding 95 % confidence interval (CI) for each category of dietary folate intake; and 6) reported the numbers of cases and/or person-years in each category (for dose‒response analyses). For duplicate publications from the same cohort project, we included those with a greater number of patients and longer follow-up. We excluded the following: 1) had randomized clinical trials, reviews and meta-analyses, letters, editorials, comments, cross-sectional studies, case‒controls, case reports, or ecological studies; 2) had nonhuman studies; 3) evaluated serum folate or folate supplementation; 4) had other GI cancers; and 5) evaluated patients with cancer at the beginning of the study or recurrence of the disease.

### 2.3 Data extraction

Two independent investigators (M.K.S. and R.K.M.) extracted the following data from the eligible studies: study characteristics (first author's name, year of publication, study location, study population), sex, age, study design (prospective cohort, nested case, and control–case cohort), study duration (follow-up), percentage of follow-up rate, number of participants/cases, number of people/year, dietary assessment method, folate dose categories, site of colorectal cancer, effect size, fully adjusted risk estimates and 95 % CIs, and list of potential confounders. Most covariate adjustment models were selected and included in the meta-analysis.

### 2.4 Quality assessment

The 9-point Newcastle–Ottawa Scale was used for the quality assessment of the included studies [28]. Studies were rated 1–3, 4–6, or 7–9 points as poor, fair, or high quality, respectively. Furthermore, GRADE was used for judgment about the quality of meta-evidence, which was graded as low for observational studies; downgraded for study limitations, inconsistency, indirectness, imprecision, and publication bias; and upgraded for large effect size, dose–response gradient, and attenuation by plausible confounding [29].

### 2.5 Statistical analysis

The RR and 95 % CI were considered the effect sizes of all the studies. The HR and OR were considered equal to the RR. We converted the per SD increase risk estimates to relative risks for the comparison of the top versus bottom third of folate intake if studies reported relative risks of mortality per 1 standard deviation (SD) increase in folate intake. We used a random effects model to calculate the overall relative risk of incidence for patients taking different dosages of folate. Cases and person-years distribution was estimated by dividing the total number of cases or person-years by the number of categories in studies that did not report. The Cochran's Q test and the I^2^ statistic were used for the assessment of heterogeneity. For the I^2^ statistics, we considered values of <25 %, 25–50 %, 50–75 %, and >75 % as low, moderate, high, and severe heterogeneity, respectively, between studies [30]. To identify possible sources of heterogeneity, subgroup analyses were also conducted. If a study reported subgroup risk estimates stratified by sex or other variables, we initially pooled the subgroup estimates using a fixed effects model and then included the resulting pooled risk estimate in the main meta-analysis. Egger's linear regression test was used for the determination of publication bias [31]. Sensitivity analysis was conducted with a random effects model by excluding each study that impacted the overall estimate and assessing the reliance on the overall effect size.

We used the generalized least squares trend estimation method for linear dose‒response analysis. When folate intake was reported as a range, we were calculating the mean of the lower and upper bounds to estimate the midpoint in each category. If the highest category was open ended, the width of the adjacent interval was assumed to be the same as that of the open-ended interval. We also examined a possible nonlinear dose‒response association using restricted cubic splines with three knots at centiles of 10 %, 50 %, and 90 % of the distribution. Statistical analyses were conducted using STATA version 14.0. A P value < 0.05 was considered to indicate statistical significance.

## 3 Results

**Fig. 1:**
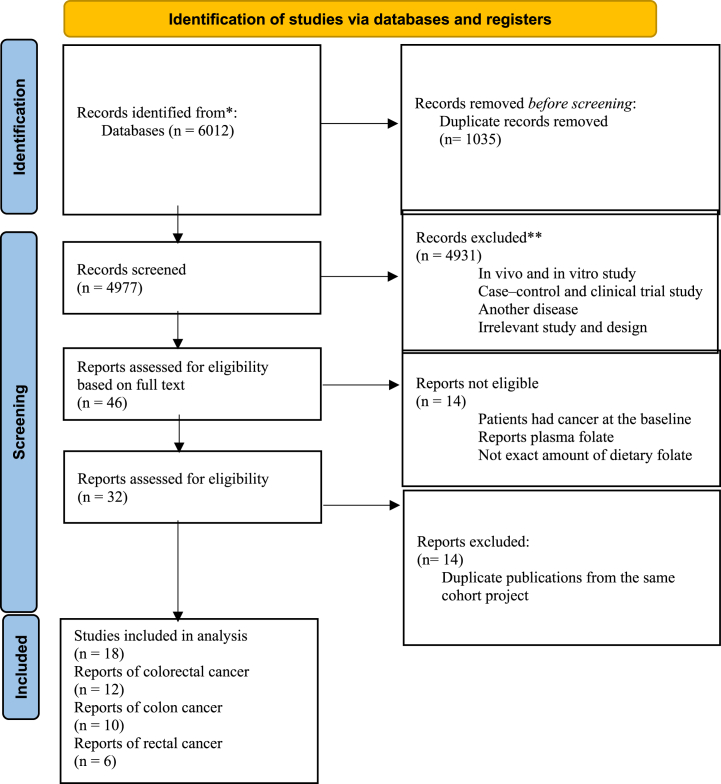
Summary of the screening and selection process of trials included in the meta-analysis.

Of the 6012 articles identified in the initial search, 46 cohort studies were assessed for eligibility based on the full text. Finally, after excluding 28 studies since the patients had cancer at baseline, reported plasma folate levels, did not report the exact amount of dietary folate, and had duplicate publications from the same cohort project, 18 prospective cohort studies were analyzed (Fig. 1).

### 3.1 Characteristics of the study cohorts

Studies were published from 1995 to 2021. The 13 cohorts were female [[32], [33], [34], [35], [36], [37], [38], [39], [40], [41], [42], [43], [44]], 4 were male [36,37,43,45], and 4 were both sexes [[46], [47], [48], [49]], with ages ranging between 25 and 93 years. In total, 931,469 participants contributed to the cohort studies, 14,860 of whom were identified as CRC cases [[32], [33], [34], [35], [36],[38], [39], [40], [41],[46], [47], [48]], 3536 as colon cancer (CC) cases [34,37,38,[42], [43], [44], [45], [46], [47], [48]], and 1075 as rectal cancer (RC) cases [34,37,[42], [43], [44],^47^]. Nine studies were conducted in the USA [32,33,[39], [40], [41],[44], [45], [46],^49^], three in Canada [35,42,48], three in the Netherlands [36,37,43], one in Australia [47], one in Sweden [34], and one in China [38]. All the studies assessed diet via food frequency questionnaires (FFQs), except one study that used 24-h recalls [49]. Most of the studies were adjusted for age, sex, body mass index (BMI), smoking status, alcohol consumption, physical activity, energy, dietary calcium, red meat, fiber, and aspirin intake. The range of folate intake reported in the cohort studies included in this meta-analysis was 40–1595 μg/d. More details are shown in Table 1, Table 2.

**Table 1:** Summary characteristics of prospective cohort studies of dietary folate intake and risk of colorectal, colon, and rectal cancer included in the systematic review and meta-analysis.

Author, Year (ref)	Study name, country	Follow-up duration (years)	Participants: Cases, (Follow-up rate)	Gender, Age	Dietary assessment	Outcome assessment	Folate dosage	RR (95%CI)	Covariates
Colorectal Cancer									
Flood, 2002 [32]	BCDDP (USA)	8.5	45264: 490 (90 %)	Female, 40–93	62-item FFQ	Self-reported	114	1.00	Sex, Alcohol, Energy intake
							160	0.79 (0.59–1.06)	
							196	0.90 (0.68–1.19)	
							241	0.99 (0.75–1.31)	
							367	0.86 (0.65–1.13)	
Zhang, 2005 [33]	The Women's Health Study (USA)	10.1	37916: 220 (95 %)	Female, >45	131-item FFQ	Self-reported	222	1.00	Age, Sex, BMI, Alcohol, Physical activity, smoking, Energy intake, red meat, Aspirin
							266	0.62 (0.40–0.98)	
							309	0.89 (0.59–1.34)	
							357	0.83 (0.55–1.26)	
							413	0.67 (0.43–1.03)	
Larsson 2005 [34]	The Swedish Mammography Cohort (Sweden)	14.8	61433: 805 (98 %)	Female, 40-75	67-item FFQ	the National Swedish Cancer Registry	136	1.00	Age, Sex, BMI, Alcohol, Smoking, Energy intake, Meat, Calcium, Fiber
							160	0.87 (0.70–1.09)	
							178	0.83 (0.64–1.06)	
							198	0.73 (0.56–0.95)	
							234	0.80 (0.60–1.06)	
Kabat 2008 [35]	NBSS (Canada)	16.4	49654: 617 (100 %)	Female, 40–59	86-item FFQ	the Canadian Cancer Database and the National Mortality Database	215	1.00	Age, Sex, BMI, Alcohol, Smoking, Energy intake
							259	0.99 (0.76–1.29)	
							301	1.09 (0.84–1.41)	
							348	1.12 (0.86–1.44)	
							397	0.89 (0.68–1.17)	
Vogel 2008 [36]	NLCS (Netherlands)	13.3	2078: 960 (100 %)	Female, 55–69	150-item FFQ	the Netherlands Cancer Registry	139	1.00	Age, Sex, BMI, Alcohol, Smoking, Physical activity, Energy intake, Meat, Calcium, Fiber
							166	0.98 (0.75–1.28)	
							188	0.95 (0.71–1.27)	
							213	1.17 (0.87–1.58)	
							267	1.25 (0.89–1.76)	
Vogel 2008 [36]	NLCS (Netherlands)	13.3	2090: 1389 (100 %)	Male, 55–69	150-item FFQ	The Netherlands Cancer Registry	161	1.00	Age, Sex, BMI, Alcohol, Smoking, Physical activity, Energy intake, Meat, Calcium, Fiber
							190	0.90 (0.71–1.13)	
							212	0.85 (0.66–1.09)	
							241	0.86 (0.66–1.11)	
							297	0.87 (0.65–1.15)	
Shrubsole 2009 [38]	The Shanghai Women's Health Study (China)	6	75221: 394 (92.7 %)	Female, 40–70	Validated FFQ	The Shanghai Cancer Registry	213	1.00	Age, Sex, BMI, Alcohol, Smoking, Physical activity, Energy intake, Meat, Calcium, Fiber, Aspirin
							235	1.30 (1.00–1.80)	
							269	1.00 (0.70–1.40)	
							318	1.20 (0.80–1.70)	
							419	1.10 (0.80–1.70)	
Gibson 2011 [46]	the NIH-AARP Diet and Health Study (USA)	9.1	214483: 6484 (90 %)	Both, 50–71	124-item FFQ	The Social Security Administration Death Master File and the cancer registries.	150	1.00	Sex, BMI, Alcohol, Smoking, Physical activity, Meat, Calcium, Aspirin
							250	0.92 (0.80–1.06)	
							350	0.86 (0.75–0.98)	
							450	0.81 (0.70–0.93)	
							550	0.86 (0.74–1.00)	
							650	0.81 (0.67–0.97)	
Razzak 2012 [39]	IWHS (USA)	13	35216: 1298 (42 %)	Female, 55–69	126-item FFQ	The Iowa Cancer Registry	201	1.00	Age, Sex, BMI, Alcohol, Smoking, Physical activity, Energy intake, Meat, Calcium
							301	1.04 (0.88–1.24)	
							462	0.91 (0.74–1.10)	
							685	0.95 (0.76–1.20)	
Zschäbitz 2013 [40]	WHI-OS (USA)	11	47028: 631 (96 %)	Female, 50–79	122-item FFQ	Self-reported	158	1.00	Age, Sex, BMI, Alcohol, Smoking, Physical activity
							221	0.86 (0.71–1.05)	
							298	0.96 (0.80–1.17)	
							388	0.83 (0.68–1.01)	
Basset 2013 [47]	The Melbourne Collaborative Cohort Study (Australia)	15.8	37109: 910 (87.7 %)	Both, 27–80	121-item FFQ	the Victorian Cancer Registry	212	1.00	Sex, Alcohol, Smoking, Physical activity, Meat, Fiber
							269	1.21 (0.98–1.48)	
							316	1.07 (0.86–1.33)	
							363	1.13 (0.91–1.40)	
							445	1.08 (0.86–1.35)	
Arthur 2019 [48]	Canadian Study of Diet, Lifestyle, and Health (Canada)	12.2	3107: 202 (100 %)	Both,	166-item FFQ	The Canadian Cancer Registry	343	1.00	Age, Sex, BMI, Alcohol, Smoking, Physical activity, Energy intake, Meat, Calcium
							440	0.92 (0.54–1.58)	
							554	1.39 (0.83–2.34)	
							685	0.81 (0.39–1.67)	
Wang 2021 [41]	NHS (USA)	28	86320: 460 (90 %)	Female, 30–55	61-item FFQ	Self-reported information	190	1.00	Age, Sex, BMI, Alcohol, Smoking, Physical activity, Energy intake, Meat, Calcium, Fiber, Aspirin
							261	0.97 (0.72–1.32)	
							332	1.16 (0.85–1.57)	
							330	1.10 (0.79–1.53)	
							329	1.15 (0.80–1.67)	
Colon Cancer									
Giovannucci, 1995 [45]	The Health Professionals Follow-up Study (USA)	6	33829: 150 (94 %) (Nonusers of aspirin)	Male, 40–75	131-item FFQ	Medical records	237	1.00	Age, Sex, BMI, Alcohol, Smoking, Physical activity, Energy intake, Meat
							302	1.13 (0.69–1.85)	
							377	0.74 (0.42–1.30)	
							533	1.31 (0.80–2.14)	
							760	0.86 (0.50–1.47)	
Giovannucci, 1995 [45]	The Health Professionals Follow-up Study (USA)	6	33829: 150 (94 %) (Aspirin user)	Male, 40–75	131-item FFQ	Medical records	237	1.00	Age, Sex, BMI, Alcohol, Smoking, Physical activity, Energy intake, Meat
							302	1.16 (0.47–2.83)	
							377	1.83 (0.80–4.18)	
							533	0.79 (0.30–2.10)	
							760	0.82 (0.33–2.08)	
SU 2001 [49]	NHANES I (USA)	20	10011: 219 (92.2 %)	Both, 25–74	24-h recalls	the National Center for Health Statistics	74	1.00	Age, Sex, BMI, Alcohol, Energy intake
							133	0.90 (0.61–1.33)	
							206	0.78 (0.51–1.33)	
							292	0.57 (0.34–0.97)	
TERRY 2002 [42]	NBSS (Canada)	10.4	56837: 200 (100 %)	Female, 40–59	86-item FFQ	The National Canadian Cancer Database	204	1.00	Age, Sex, BMI, Alcohol, Smoking, Physical activity Energy intake
							256	0.50 (0.30–1.00)	
							296	0.80 (0.50–1.40)	
							338	0.80 (0.40–1.40)	
							416	0.60 (0.30–1.10)	
Harnack 2002 [44]	IWHS (USA)	13	41836: 598 (42.5 %)	Female, 55–69	127-item FFQ	The State Health Registry of Iowa	132	1.00	Age, Sex, BMI, Alcohol, Smoking, Energy intake, Calcium
							269	1.04 (0.80–1.35)	
							357	0.96 (0.71–1.29)	
							520	0.92 (0.65–1.29)	
							1595	1.12 (0.77–1.63)	
Vogel 2006 [37]	NLCS (Netherlands)	7.3	2136: 186 (100 %)	Female, 55–69	150-item FFQ	The Netherlands Cancer Registry	143	1.00	Age, Sex, BMI, Alcohol, Smoking, Energy intake, fiber
							187	0.94 (0.61–1.44)	
							248	0.82 (0.45–1.49)	
Vogel 2006 [37]	NLCS (Netherlands)	7.3	2040: 213 (100 %)	Male, 55–69	150-item FFQ	The Netherlands Cancer Registry	163	1.00	Age, Sex, BMI, Alcohol, Smoking, Energy intake, fiber
							211	0.69 (0.46–1.02)	
							280	0.96 (0.61–1.54)	
Brink 2005 [43]	NLCS (Netherlands)	2.3	3346: 199 (100 %)	Female, 55–69	150-item FFQ	The Netherlands Cancer Registry	Linear	Linear	Age, Sex, BMI, Alcohol, Smoking, Physical activity, Energy intake, Meat, Fiber
Brink 2005 [43]	NLCS (Netherlands)	2.3	3346: 231 (100 %)	Male, 55–69	150-item FFQ	The Netherlands Cancer Registry	Linear	Linear	Age, Sex, BMI, Alcohol, Smoking, Physical activity, Energy intake, Meat, Fiber
Larsson 2005 [34]	The Swedish Mammography Cohort (Sweden)	14.8	61433: 419 (98 %)	Female, 40-75	67-item FFQ	the National Swedish Cancer Registry	136	1.00	Age, Sex, BMI, Alcohol, Smoking, Energy intake, Meat, Calcium, Fiber
							160	0.81 (0.60–1.10)	
							178	0.72 (0.51–1.02)	
							198	0.67 (0.47–0.96)	
							234	0.61 (0.41–0.91)	
Schernhammer 2011 [50]	NHS (USA)	22	88691: 375 (58 %)	Female, 30–55	61-item FFQ	Self-reported	150	1.00	Age, Sex, BMI, Alcohol, Smoking, Physical activity, Energy intake, Meat, Calcium, Aspirin
							250	0.81 (0.62–1.06)	
							350	0.80 (0.57–1.12)	
							450	0.78 (0.59–1.05)	
Basset 2013 [47]	The Melbourne Collaborative Cohort Study (Australia)	15.8	37109: 581 (87.7 %)	Both, 27–80	121-item FFQ	the Victorian Cancer Registry	212	1.00	Sex, Alcohol, Smoking, Physical activity, Meat, Fiber
							269	1.15 (0.88–1.49)	
							316	1.09 (0.84–1.43)	
							363	1.07 (0.82–1.40)	
							445	0.98 (0.74–1.31)	
Arthur 2019 [48]	Canadian Study of Diet, Lifestyle, and Health (Canada)	12.2	3107: 165 (100 %)	Both, ?	166-item FFQ	The Canadian Cancer Registry	343	1.00	Age, Sex, BMI, Alcohol, Smoking, Physical activity, Energy intake, Meat, Calcium
							440	0.93 (0.64–1.36)	
							554	1.14 (0.78–1.68)	
							685	0.96 (0.58–1.58)	
Rectal Cancer									
TERRY 2002 [42]	NBSS (Canada)	10.4	56837: 95 (100 %)	Female, 40–59	86-item FFQ	The National Canadian Cancer Database	204	1.00	Age, Sex, BMI, Alcohol, Smoking, Physical activity Energy intake
							256	1.10 (0.60–2.30)	
							296	1.20 (0.60–2.50)	
							338	0.90 (0.40–2.00)	
							416	0.70 (0.30–1.80)	
Harnack 2002 [44]	IWHS (USA)	13	41836: 123 (42.5 %)	Female, 55–69	127-item FFQ	The State Health Registry of Iowa	157	1.00	Age, Sex, BMI, Alcohol, Smoking, Energy intake, Calcium
							373	0.82 (0.52–1.29)	
							1509	0.89 (0.52–1.51)	
Brink 2005 [43]	NLCS (Netherlands)	2.3	3346: 51 (100 %)	Female, 55–69	150-item FFQ	The Netherlands Cancer Registry	Linear	Linear	Age, Sex, BMI, Alcohol, Smoking, Physical activity, Energy intake, Meat, Fiber
Brink 2005 [43]	NLCS (Netherlands)	2.3	3346: 99 (100 %)	Male, 55–69	150-item FFQ	The Netherlands Cancer Registry	Linear	Linear	Age, Sex, BMI, Alcohol, Smoking, Physical activity, Energy intake, Meat, Fiber
Larsson 2005 [34]	The Swedish Mammography Cohort (Sweden)	14.8	61,433: 252 (98 %)	Female, 40-75	67-item FFQ	the National Swedish Cancer Registry	136	1.00	Age, Sex, BMI, Alcohol, Smoking, Energy intake, Meat, Calcium, Fiber
							160	0.81 (0.53–1.23)	
							178	0.85 (0.54–1.33)	
							198	0.82 (0.51–1.32)	
							234	0.93 (0.55–1.56)	
Vogel 2006 [37]	NLCS (Netherlands)	7.3	2136: 45 (100 %)	Female, 55–69	150-item FFQ	The Netherlands Cancer Registry	143	1.00	Age, Sex, BMI, Alcohol, Smoking, Energy intake, fiber
							187	1.71 (0.72–4.04)	
							248	1.54 (0.55–4.33)	
Vogel 2006 [37]	NLCS (Netherlands)	7.3	2040: 84 (100 %)	Male, 55–69	150-item FFQ	The Netherlands Cancer Registry	163	1.00	Age, Sex, BMI, Alcohol, Smoking, Energy intake, fiber
							211	1.11 (0.63–1.97)	
							280	0.91 (0.41–2.01)	
Basset 2013 [47]	The Melbourne Collaborative Cohort Study (Australia)	15.8	37109: 326 (87.7 %)	Both, 27–80	121-item FFQ	the Victorian Cancer Registry	212	1.00	Sex, Alcohol, Smoking, Physical activity, Meat, Fiber
							269	1.32 (0.94–1.85)	
							316	1.00 (0.69–1.44)	
							363	1.25 (0.88–1.79)	
							445	1.26 (0.87–1.83)	

Abbreviations: BCDDP: Breast Cancer Detection Demonstration Project; BMI: Body Mass Index; FFQ: Food Frequency Questionnaire; IWHS: The Iowa Women's Health Study; NBSS: National Breast Screening Study; NHANES: National Health and Nutrition Examination Survey; NHS: National Health Service; NLCS: Netherlands Cohort Study; NIH-AARP: National Institutes of Health-American Association of Retired Persons; WHI-OS: The Women's Health Initiative Observational Study.

**Table 2:** Summary characteristics of prospective cohort studies of total folate intake and risk of colorectal, colon, and rectal cancer included in the systematic review and meta-analysis.

Author, Year (ref)	Study name, country	Follow-up duration (years)	Participants: Cases, (Follow-up rate)	Gender, Age	Dietary assessment	Outcome assessment	Folate dosage	RR (95%CI)	Covariates
Colorectal Cancer									
Flood**,** 2002 [32]	BCDDP (USA)	8.5	45264: 490 (90 %)	Female, 40–93	62-item FFQ	Self-reported	156	1.00	Sex, Alcohol, Energy intake
							221	0.94 (0.7–1.25)	
							314	0.89 (0.66–1.20)	
							504	0.97 (0.73–1.30)	
							762	1.01 (0.75–1.35)	
Zhang, 2005 [33]	The Women's Health Study (USA)	10.1	37916: 220 95 %	Female, >45	131-item FFQ	Self-reported	230	1.00	Age, Sex, BMI, Alcohol, Physical activity, smoking, Energy intake, Red meat, Aspirin
							288	1.10 (0.71–1.70)	
							355	0.91 (0.58–1.44)	
							503	0.97 (0.62–1.52)	
							725	1.16 (0.76–1.79)	
Gibson 2011 [46]	the NIH-AARP Diet and Health Study (USA)	9.1	214483: 6484 (90 %)	Both, 50–71	124-item FFQ	the Social Security Administration Death Master File and the cancer registries.	150	1.00	Sex, BMI, Alcohol, Smoking, Physical activity, Meat, Calcium, Aspirin
							250	0.90 (0.76–1.08)	
							350	0.82 (0.69–0.98)	
							450	0.75 (0.63–0.90)	
							550	0.75 (0.62–0.90)	
							650	0.77 (0.64–0.92)	
							750	0.75 (0.63–0.89)	
							850	0.75 (0.62–0.90)	
							950	0.70 (0.58–0.84)	
Zschäbitz 2013 [40]	WHI-OS (USA)	11	47028: 808 (96 %)	Female, 50–79	122-item FFQ	Self-reported	92	1.00	Age, Sex, BMI, Alcohol, Smoking, Physical activity
							392	0.92 (0.76–1.12)	
							741	0.96 (0.79–1.16)	
							1138	0.90 (0.74–1.10)	
Wang 2021 [41]	NHS (USA)	28	86320: 460 (90 %)	Female, 30–55	61-item FFQ	Self-reported information	189	1.00	Age, Sex, BMI, Alcohol, Smoking, Physical activity, Energy intake, Meat, Calcium, Fiber, Aspirin
							283	1.12 (0.84–1.49)	
							377	1.02 (0.74–1.39)	
							751	1.13 (0.81–1.58)	
							1126	0.90 (0.59–1.38)	

Abbreviations: BCDDP: Breast Cancer Detection Demonstration Project; BMI: Body Mass Index; FFQ: Food Frequency Questionnaire; NHS: National Health Service; NIH-AARP: National Institutes of Health-American Association of Retired Persons; WHI-OS: The Women's Health Initiative Observational Study.

### 3.2 Colorectal cancer

**Fig. 2:**
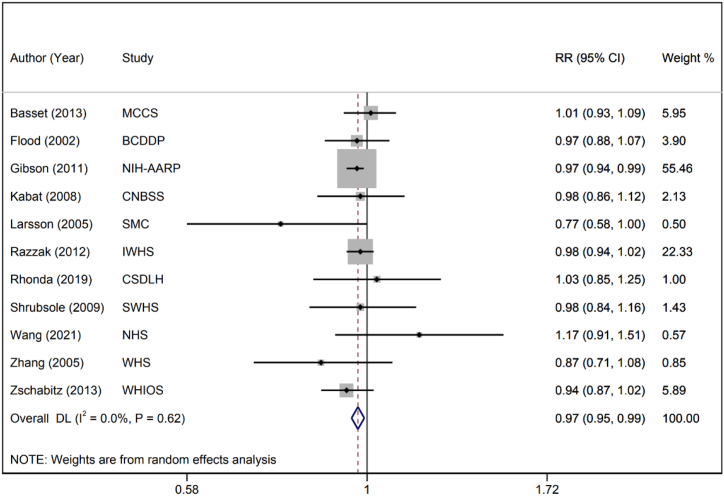
Forest plot for linear dose‒response analysis between dietary folate intake and risk of colorectal cancer.

Twelve publications evaluated the association between dietary folate intake and risk of CRC [[32], [33], [34], [35], [36],[38], [39], [40], [41],[46], [47], [48]]. These studies had a total of 696,919 participants and 14,869 cases of CRC. The summary RR per 100 μg/day of dietary folate was 0.97 (95 % CI: 0.95–0.99, I^2^: 0.0 %, P-heterogeneity = 0.616), indicating a significant linear inverse association between folate intake and the risk of CRC (Fig. 2). This difference was related to BMI (0.97 (95 % CI: 0.95–0.99)), and a more protective effect was observed for subjects who drank alcohol (0.97 (95 % CI: 0.95–0.99)) and those who smoked (0.97 (95 % CI: 0.95–0.99)). Additionally, a significant relationship was detected in studies not adjusted for fiber intake (0.97 (95 % CI: 0.95–0.99)). However, dietary folate effectiveness was not dependent on sex, age, daily intake of total energy, red meat, calcium intake, or aspirin intake (Table 3). According to the nonlinear dose‒response analysis, there was no significant association between dietary folate intake and the risk of CRC (P nonlinearity = 0.738) (Supplementary Fig. 2). Similarly, overall results, the analysis of women did not reveal a significant nonlinear association (P nonlinearity = 0.957) (Supplementary Fig. 3).

**Table 3:** Relative risk of 100 μg/d increment in folate intake.

Comparison		n	Pooled RRs	I^2^ (%)	P-heterogeneity	P-between
*Colorectal Cancer (dietary folate)*						
All studies		11	0.97 (0.95–0.99)	0.0 %	0.616	0.005
Sex						
Both		2	0.97 (0.95–1.00)	0.0 %	0.325	0.036
Women		9	0.97 (0.94–1.00)	0.0 %	0.521	0.064
Study Design						
Prospective cohort study		10	0.97 (0.95–0.99)	0.0 %	0.555	0.004
Case-cohort study		1	1.03 (0.85–1.25)	–	–	0.764
Adjustments						
Age	Yes	5	0.97 (0.94–1.01)	2.0 %	0.414	0.089
	No	3	0.97 (0.95–1.00)	0.0 %	0.614	0.029
BMI	Yes	9	0.97 (0.95–0.99)	0.0 %	0.521	0.004
	No	2	0.99 (0.93–1.06)	0.0 %	0.515	0.868
Alcohol	Yes	11	0.97 (0.95–0.99)	0.0 %	0.616	0.005
	No	–	–	–	–	–
Physical	Yes	8	0.97 (0.95–0.99)	0.0 %	0.626	0.010
activity	No	3	0.95 (0.86–1.04)	24.4 %	0.267	0.279
Smoking	Yes	9	0.97 (0.95–0.99)	0.0 %	0.727	0.009
	No	2	0.90 (0.72–1.11)	59.1 %	0.118	0.318
Energy	Yes	8	0.98 (0.94–1.01)	0.0 %	0.495	0.171
	No	3	0.97 (0.95–0.99)	0.0 %	0.435	0.014
Red meat	Yes	8	0.98 (0.95–1.00)	4.9 %	0.393	0.040
	No	3	0.96 (0.91–1.01)	0.0 %	0.822	0.122
Fiber	Yes	4	0.99 (0.88–1.10)	41.8 %	0.161	0.842
	No	7	0.97 (0.95–0.99)	0.0 %	0.893	0.003
Calcium	Yes	6	0.97 (0.95–1.00)	7.1 %	0.371	0.046
	No	5	0.97 (0.93–1.01)	0.0 %	0.604	0.176
Aspirin	Yes	4	0.97 (0.93–1.01)	4.8 %	0.369	0.139
	No	7	0.98 (0.95–1.01)	0.0 %	0.556	0.112
*Colorectal Cancer (Total folate)*						
All studies		5	0.98 (0.97–0.99)	0.0 %	0.427	<0.001
Sex						
Both		1	0.98 (0.97–0.99)	–	–	<0.001
Women		4	0.99 (0.98–1.01)	0.0 %	0.537	0.395
Adjustments						
Age	Yes	3	0.99 (0.97–1.01)	0.0 %	0.524	0.243
	No	2	0.99 (0.96–1.01)	52.1 %	0.149	0.403
BMI	Yes	4	0.98 (0.97–0.99)	0.0 %	0.568	<0.001
	No	1	1.01 (0.97–1.05)	–	–	0.623
Alcohol	Yes	5	0.98 (0.97–0.99)	0.0 %	0.427	<0.001
	No	–	–	–	–	–
Physical activity	Yes	4	0.98 (0.97–0.99)	0.0 %	0.568	<0.001
	No	1	1.01 (0.97–1.05)	–	–	0.623
Smoking	Yes	4	0.98 (0.97–0.99)	0.0 %	0.568	<0.001
	No	1	1.01 (0.97–1.05)	–	–	0.623
Energy	Yes	3	1.00 (0.97–1.03)	0.0 %	0.378	0.939
	No	2	0.98 (0.97–0.99)	0.0 %	0.379	<0.001
Red meat	Yes	3	0.98 (0.97–0.99)	0.0 %	0.530	<0.001
	No	2	0.99 (0.98–1.01)	0.0 %	0.378	0.519
Fiber	Yes	1	0.97 (0.91–1.02)	–	–	0.227
	No	4	0.99 (0.97–1.00)	12.8 %	0.328	0.010
Calcium	Yes	2	0.98 (0.97–0.99)	0.0 %	0.618	<0.001
	No	3	0.99 (0.98–1.01)	0.0 %	0.556	0.606
Aspirin	Yes	3	0.98 (0.97–0.99)	0.0 %	0.530	<0.001
	No	2	0.99 (0.98–1.01)	0.0 %	0.378	0.519
*Colon Cancer (dietary folate)*						
All studies		8	0.93 (0.87–0.99)	33.7 %	0.159	0.029
Sex						
Both		2	0.89 (0.72–1.11)	69.9 %	0.068	0.304
Men		2	0.96 (0.89–1.04)	0.0 %	0.455	0.327
Women		5	0.92 (0.82–1.03)	38.7 %	0.163	0.131
Study Design						
Prospective cohort study		5	0.92 (0.85–1.00)	52.8 %	0.076	0.044
Case-cohort study		3	0.96 (0.86–1.08)	0.0 %	0.384	0.526
Adjustments						
Age	Yes	7	0.92 (0.85–0.99)	39.9 %	0.125	0.026
	No	1	0.98 (0.88–1.08)	–	–	0.647
BMI	Yes	6	0.94 (0.87–1.01)	30.9 %	0.204	0.075
	No	2	0.89 (0.72–1.11)	69.9 %	0.068	0.304
Alcohol	Yes	8	0.93 (0.87–0.99)	33.7 %	0.159	0.029
	No	–	–	–	–	–
Physical	Yes	6	0.96 (0.92–1.01)	0.0 %	0.766	0.088
activity	No	2	0.74 (0.62–0.89)	0.0 %	0.405	0.001
Smoking	Yes	6	0.96 (0.92–1.01)	0.0 %	0.766	0.088
	No	2	0.74 (0.62–0.89)	0.0 %	0.405	0.001
Energy	Yes	7	0.92 (0.85–0.99)	39.9 %	0.125	0.026
	No	1	0.98 (0.88–1.08)	–	–	0.647
Red meat	Yes	6	0.95 (0.90–1.01)	26.8 %	0.234	0.105
	No	2	0.80 (0.67–0.96)	0.0 %	0.692	0.014
Fiber	Yes	3	0.87 (0.71–1.06)	62.3 %	0.070	0.176
	No	5	0.94 (0.88–1.01)	23.3 %	0.266	0.077
Calcium	Yes	3	0.91 (0.78–1.06)	66.5 %	0.051	0.245
	No	5	0.94 (0.88–1.01)	12.2 %	0.336	0.078
Aspirin	Yes	1	0.93 (0.85–1.02)	–	–	0.119
	No	8	0.93 (0.87–1.00)	37.0 %	0.134	0.052
*Rectal Cancer (dietary folate)*						
All studies		5	0.98 (0.90–1.08)	16.6 %	0.309	0.728

*P-value <0.05 was significant.

Abbreviation: BMI: body mass index; RR: relative risk.

Five cohorts [32,33,40,41,46] with a total of 431,011 participants and 8462 cases were investigated for the effect of total folate on CRC risk. The summary RR per 100 μg/day total folate was 0.98 (95 % CI: 0.97–0.99, I^2^: 0.0 %, P-heterogeneity = 0.427) (Supplementary Fig. 1). Additionally, we found greater effectiveness of total folate intake on CRC incidence in studies adjusted for BMI, alcohol consumption, smoking status, physical activity, red meat intake, calcium intake, and aspirin use (for all mentioned variables: 0.98 (95 % CI: 0.97–0.99)) (Table 3). Overall and female nonlinear dose‒response analyses did not reveal a significant relationship between total folate intake and the risk of CRC (P nonlinearity = 0.390 and P nonlinearity = 0.992, respectively) (Supplementary Figs. 2 and 3).

### 3.3 Colon cancer

**Fig. 3:**
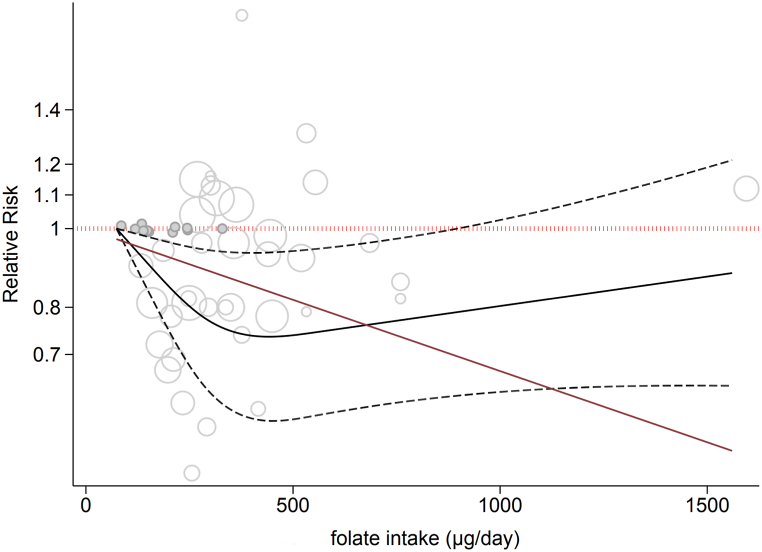
Nonlinear dose‒response analysis between dietary folate intake and risk of colon cancer.

Ten prospective cohort studies [34,37,[42], [43], [44], [45],[47], [48], [49], [50]] evaluating the association between dietary folate intake and the risk of CC were included in the dose‒response meta-analyses. The studies included 340,375 participants with 3536 cases. Analysis of the data showed that each 100 μg/day dietary folate treatment was positively related to a 7 % lower risk of CC (0.93 (95 % CI = 0.87–0.99, I^2^ = 33.7 %, P-heterogeneity = 0.159) (Supplementary Fig. 4). This effect did not depend on sex. In addition, a significant relationship was detected after adjusting for age, alcohol consumption, and energy intake (Table 2). There was evidence of nonlinearity in which up to 500 μg/day dietary folate intake was inversely associated with CC (P nonlinearity = 0.04) (Fig. 3). However, subgroup analysis revealed that the association was significant for females (P nonlinearity for females = 0.03; P nonlinearity for males = 0.61) (Supplementary Fig. 5).

### 3.4 Rectal cancer

The results of linear dose‒response analysis of six studies [34,37,[42], [43], [44],^47^] with 204,737 participants and 1075 RC patients showed that each 100 μg/day dietary folate did not have a significant effect on the risk of RC (0.98 (95 % CI: 0.90–1.08), I^2^: 16.6 %, P-heterogeneity = 0.309) (Supplementary Fig. 6). Additionally, in the nonlinear analysis, we did not observe a significant relationship between dietary folate and RC in general or in females (P nonlinearity = 0.94 and = 0.98, respectively) (Supplementary Fig. 7).

### 3.5 Publication bias and sensitivity analyses

Using Egger's linear regression test to assess publication bias revealed some evidence for publication bias for colon cancer.

According to the sensitivity analyses based on a random effects model, when Larsson et al. [34] and SU et al. [49] excluded from the analysis for colon studies, the results changed to 0.960 and 95 % CI = 0.92–1.01 (no significant association) without heterogeneity (I^2^ = 0.0 %, P = 0.088).

### 3.6 Quality assessment

According to the bias assessment of the Newcastle–Ottawa Scale, the quality of all the studies was considered good (Supplementary Table 2). Based on the GRADE approach, the certainty of evidence was rated moderate for CRC (dietary folate and total folate), and it was rated low for CC and RC due to downgrades for imprecision (Supplementary Table 3).

## 4 Discussion

In recent decades, a combination of evidence has suggested that folate may play a crucial role in the preventing and developing of malignancies, including GI cancers [12,51,52]. Our dose-response meta-analysis revealed that each 100-μg increase in dietary folate intake has an inverse association with CRC incidence. This approach can be more effective for individuals who have a high BMI, drink alcohol, or smoke. Additionally, the nonlinear analysis showed a significant inverse relationship between dietary folate intake (up to 500 μg/day) and CC, although subgroup analysis indicated that this relationship can be significant in females but not in males.

Epidemiological and experimental studies have shown that folate deficiency in normal tissues increases the risk of neoplastic transformation, particularly in colonocytes, which grow quickly and require additional folate [12,53]. Similarly, to our study results, a meta-analysis of 7 cohorts and 9 case‒control studies showed that dietary folate could reduce the risk of CRC. As a result, dietary folate is associated with a lower risk than total folate [23]. In addition, another systematic review and meta-analysis of observational (case‒control and cohort) studies comparing high versus low total folate intake suggested that the summary risk estimate for higher folate intake levels reduces one of the comprehended risks associated with developing CRC [54]. This was the first study to report the findings separately in different parts of the large intestine. Another meta-analysis focused on the dose‒response association between serum folate levels and CRC incidence in observational studies showed that 10 nmol/L circulating folate had a minimal effect on reducing the risk of CRC [24,55].

Meta-analyses have also been conducted on folic acid supplementation and CRC risk. Overall, the results of these studies showed that supplementation with 0.5–2.5 mg of folic acid per day in the long term was not associated with CRC risk in the general population [24,26]. Nonetheless, another study found no increase in the risk of adenomatous lesions with 0.5–1 mg of folate taken daily for less than 3 years. However, daily supplementation with 2.5 mg of folic acid for 5 years can increase this risk [22]. It should be noted that this study had fewer publications than the other one, and the risk of incidence was reported with odds ratios, which could have some exaggerated results. Also, other studies have reached that folate supplementation four times above the daily dietary intake is associated with suppressing the development of microscopic to macroscopic tumors in colorectal tissue. Studies on rats have shown that supraphysiological doses of folate also increase colorectal tumorigenesis. However, higher folate levels did not appear to increase tumorigenesis [12,56,57].

Generally, dietary folate is correlated with fiber intake in the daily diet. Fiber-rich diets can be protective factors against colorectal cancer [58,59]. Some studies adjusted dietary fiber as a confounder. Alcohol consumption is another confounding factor with direct impacts on folate intake, bioavailability, and structural changes in the body's folate stores [60]. Most of the studies adjusted for this confounding factor or reported the risk of CRC incidence according to alcohol consumption [32,35,40,45,48,49]. Additionally, other confounding factors, including age, sex, BMI, smoking status, physical activity, energy intake, red meat intake, and calcium intake, are the main lifestyle challenges that can affect the incidence of CRC; most studies have adjusted for these confounding factors. However, this point must also be considered because our included studies were designed in developed countries (most of which were in the USA) that have different dietary patterns and lifestyles, possibly due to higher economic status, as well as because the availability of screening programs can better control the risk of CRC. However, in developing countries the incidence of CRC has been increasing because of unsuitable lifestyles, as mentioned above [1]. Prospective cohort studies in developing countries are needed to carefully examine the dosage of folate intake and the risk of CRC.

Folate is known as an essential component in the de novo biosynthesis of purines and thymidylate by modulating DNA methylation, because of that, it is a vital epigenetic component for the gene expression [61]. Even polymorphisms in the methylenetetrahydrofolate reductase (MTHFR) gene may modulate CRC risk [62]. A meta-analysis showed RRs for high against low total folate for the 677CC genotype and the 677 TT genotype, suggesting that the reduction risk of developing CRC is associated with the 677 TT genotype [21]. These studies show the important role of sufficient folate intake in preventing CRC.

The current meta-analysis is the first study that performed a dose‒response analysis and reported a protective effect of folate on the risk of CRC for each 100-μg increase in diet. Another strength of the current meta-analysis was the low heterogeneity among studies for overall incidence of colorectal, colon, and rectal cancer. Additionally, our study is the first to report this effect in different parts of the large intestine. The good quality of each study was a positive point in the included studies, although the overall strength of the evidence was low or moderate.

One of the weaknesses of the study is that folate intake was assessed via the FFQ, which can create recall bias in observational studies and under- or overestimate dietary folate intake. Because publications from the same cohort project involved the same participants, we had to exclude many studies. Additionally, all studies conducted in developed countries restrict us from generalizing the results.

The meta-analysis concluded an inverse link between dietary folate intake and colorectal cancer risk. A 100 μg/day rise in folate intake within recommended levels from rich sources may help shield against colorectal neoplasms, notably in at-risk individuals (e.g., those with higher BMI, alcohol consumers, and smokers). This analysis backs public health advice to boost folate intake for CRC prevention.

## Funding

This research received a grant from the Department of Nutrition, 10.13039/501100004484Tehran University of Medical Sciences (grant number 1400-3-212-57313).

## Availability of data and materials

The datasets used and/or analyzed during the current study are available from the corresponding author upon reasonable request.

## Ethics approval and consent to participate

Not applicable.

## CRediT authorship contribution statement

**Masoumeh Khalighi Sikaroudi:** Writing – review & editing, Writing – original draft, Validation, Resources, Project administration, Methodology, Investigation, Data curation, Conceptualization. **Sepideh Soltani:** Supervision, Software, Resources, Methodology, Investigation, Data curation. **Roya Kolahdouz-Mohammadi:** Writing – original draft, Investigation, Data curation. **Roya Imanifard:** Writing – review & editing. **Shima Abdollahi:** Writing – original draft, Validation, Methodology, Investigation. **Hossein Shahinfar:** Writing – review & editing, Writing – original draft, Data curation. **Gholamreza Mohammadi Farsani:** Writing – review & editing, Writing – original draft, Visualization, Supervision, Project administration, Methodology, Investigation, Funding acquisition, Conceptualization.

## Declaration of competing interest

The authors declare the following financial interests/personal relationships which may be considered as potential competing interests: Gholamreza Mohammadi Farsani reports financial support was provided by 10.13039/501100004484Tehran University of Medical Sciences. Gholamreza Mohammadi Farsani reports a relationship with 10.13039/501100004484Tehran University of Medical Sciences that includes: funding grants. Gholamreza Mohammadi Farsani has patent pending to 1400-3-212-57313. The authors declare that there are no conflicts of interest. If there are other authors, they declare that they have no known competing financial interests or personal relationships that could have appeared to influence the work reported in this paper.
