# Supplementary material for: The association between dietary folate intake and risk of colorectal cancer incidence: A systematic review and dose‒response meta-analysis of cohort studies
Source: Heliyon. 2024 Jun 26;10(13):e33564. doi: 10.1016/j.heliyon.2024.e33564 (PMC11279277; doi:10.1016/j.heliyon.2024.e33564)
Supplement: Multimedia component 1 [file mmc1.docx]

**The association between dietary folate intake and risk of colorectal cancer incidence**: **A systematic review and dose-response meta-analysis of cohort studies.**

Supplementary data including 3 Tables and 7 Figures

| ***Supplementary Tables legends:*** | | |
| --- | --- | --- |
| **sTable 1** | Search strategies including the key terms and the queries for each database. | **Page**:2-3 |
| **sTable 2** | Study quality assessment by a 9-point Newcastle-Ottawa Scale for cohort studies. | **Page**:4-5 |
| **sTable 3** | Meta-evidence judgment quality based on the GRADE | **Page**:6 |
| ***Supplementary Figures legends:*** | | |
| **sFigure 1** | Forest plot for linear dose-response analysis between total folate intake and risk of colorectal cancer. | **Page**:7 |
| **sFigure 2** | Non-linear dose-response analysis between dietary and total folate intake and risk of colorectal cancer. | **Page**:7 |
| **sFigure 3** | Non-linear dose-response analysis between dietary and total folate intake and risk of colorectal cancer in female. | **Page**:8 |
| **sFigure 4** | Forest plot for linear dose-response analysis between dietary folate intake and risk of colon cancer. | **Page**:8 |
| **sFigure 5** | Non-linear dose-response analysis between dietary folate intake and risk of colon cancer in female and male. | **Page**:9 |
| **sFigure 6** | Forest plot for linear dose-response analysis between dietary folate intake and risk of rectal cancer. | **Page**:9 |
| **sFigure 7** | Non-linear dose-response analysis between dietary folate intake and risk of rectal cancer in overall and females. | **Page**:10 |

**Literature search strategy**

The following databases were searched:

- PubMed/Medline
- Scopus
- ISI Web of Science

| **Supplementary Table 1.** Search strategies including the key terms and the queries for each database |
| --- |
| **1.Pubmed**  (“Folic Acid [all fields] OR “Dietary folate” [all fields] OR “Total folate”[all fields] OR Vitamin B9 [all fields] OR “Folate status”[ all fields] OR “Folic Acid”[MeSH Terms] OR "folate"[Mesh Terms] OR "Vitamin B9"[MeSH Terms] OR "vitamin B"[Mesh Terms]”)  ------------------------------------------------------------------------------------------------------------------------  (“colorectal [all fields] OR “colon”[all fields] OR “rectum”[all fields] OR bowel [all fields] OR “cancer”[ all fields] OR “Colorectal Neoplasms”[all fields] OR “colorectal cancer”[all fields] OR “colorectal Adenoma”[all fields] OR “colorectal carcinoma”[all fields] OR “colon cancer”[ all fields] OR " rectal cancer"[ all fields] OR colorectal [Mesh Terms] OR “colon”[ Mesh Terms] OR “rectum”[ Mesh Terms] OR bowel [Mesh Terms] OR “cancer”[ Mesh Terms] OR “Colorectal Neoplasms”[ Mesh Terms] OR “colorectal Adenoma”[ Mesh Terms] OR “colorectal carcinoma”[ Mesh Terms] OR “colon cancer”[MeSH Terms] OR "Rectal Neoplasms" [Mesh Terms]”)  ------------------------------------------------------------------------------------------------------------------------  (“*Observational[all fields] OR “*Follow up”[all fields] OR “*Follow-up”[all fields] OR *Prospective[all fields] OR “Nested case-control”[ all fields] OR “Relative risk”[all fields] OR “Risk ratio”[all fields] OR “Hazard ratio”[all fields] OR “odds ratio”[all fields] OR longitudinal[all fields] OR “*Cohort Studies”[MeSH Terms] OR Incidence[MeSH Terms] OR “longitudinal Studies”[MeSH Terms] OR "Epidemiologic Studies"[Mesh Terms] OR "*prospective studies"[MeSH Terms] OR "*longitudinal studies"[Mesh Terms] OR *prospectively [all fields] OR *cohort [all fields] OR *observations[all fields] OR "Cross-Sectional Studies"[Mesh Terms] OR "Cross-Sectional Study"[ all fields] OR "Case-control Studies"[Mesh Terms] OR " Case-control Study"[all fields] OR "Retrospective Studies"[Mesh Terms] OR "Retrospective Study"[all fields]” |
| **2.Scopus** |
| TITLE-ABS-KEY (Folic Acid) OR TITLE-ABS-KEY ("Dietary folate") OR TITLE-ABS-KEY ("folate") OR TITLE-ABS-KEY ("Total folate") OR TITLE-ABS-KEY (Vitamin B9) OR TITLE-ABS-KEY (“Folate status”) OR TITLE-ABS-KEY (“B vitamins”) OR TITLE-ABS-KEY (“vitamin B”)  ------------------------------------------------------------------------------------------------------------------------  TITLE-ABS-KEY (colorectal) OR TITLE-ABS-KEY ("colon") OR TITLE-ABS-KEY ("rectum") OR TITLE-ABS-KEY (bowel) OR TITLE-ABS-KEY (“cancer”) OR TITLE-ABS-KEY (“Colorectal Neoplasms”) OR TITLE-ABS-KEY (“colorectal cancer”) OR TITLE-ABS-KEY (“colorectal Adenoma”) OR TITLE-ABS-KEY (“colorectal carcinoma”) OR TITLE-ABS-KEY (colon cancer) OR TITLE-ABS-KEY (rectal cancer) OR TITLE-ABS-KEY (rectal Neoplasms)  ------------------------------------------------------------------------------------------------------------------------  TITLE-ABS-KEY (*Observational) OR TITLE-ABS-KEY ("*Follow up") OR TITLE-ABS-KEY ("*Follow-up") OR TITLE-ABS-KEY (*Prospective) OR TITLE-ABS-KEY (“Nested case-control”) OR TITLE-ABS-KEY (“Relative risk”) OR TITLE-ABS-KEY (“Risk ratio”) OR TITLE-ABS-KEY (“Hazard ratio”) OR TITLE-ABS-KEY (“odds ratio”) OR TITLE-ABS-KEY (longitudinal) OR TITLE-ABS-KEY (*prospectively) OR TITLE-ABS-KEY (*cohort) OR TITLE-ABS-KEY (*observations) OR TITLE-ABS-KEY ("Cross-Sectional Study") OR TITLE-ABS-KEY ("Case-control Study") OR TITLE-ABS-KEY ("Retrospective Studies") OR TITLE-ABS-KEY ("Retrospective Study") |
| **3. ISI WOS** |
| TI= ("Folic Acid*") OR TI= ("Dietary folate") OR TI= ("Total folate") OR TI= (folate") OR TI= ("Vitamin B9") OR TI= ("Folate status") OR TI= ("B vitamins") OR TI= ("vitamin B") OR AB= ("Folic Acid*") OR AB= ("Dietary folate") OR AB= ("Total folate") OR AB= (folate") OR AB= ("Vitamin B9") OR AB= ("Folate status") OR AB= ("B vitamins") OR AB= ("vitamin B") OR KW= ("Folic Acid*") OR KW= ("Dietary folate") OR KW= ("Total folate") OR KW= (folate") OR KW= ("Vitamin B9") OR KW= ("Folate status") OR KW= ("B vitamins") OR KW= ("vitamin B")  AND  TI= ("colorectal") OR TI= ("colon") OR TI= ("rectum") OR TI= ("bowel") OR TI= ("cancer") OR TI= ("Colorectal Neoplasms") OR TI= ("colorectal cancer") OR TI= ("colorectal Adenoma") OR TI= ("colorectal carcinoma") OR TI= ("colon cancer") OR TI= ("rectal cancer") OR TI= ("rectal Neoplasms") OR AB= ("colorectal") OR AB= ("colon") OR AB= ("rectum") OR AB= ("bowel") OR AB= ("cancer") OR AB= ("Colorectal Neoplasms") OR AB= ("colorectal cancer") OR AB= ("colorectal Adenoma") OR AB= ("colorectal carcinoma") OR AB= ("colon cancer") OR AB= ("rectal cancer") OR AB= ("rectal Neoplasms") OR KW= ("colorectal*") OR KW= ("colon") OR KW= ("rectum") OR KW= ("bowel") OR KW= ("cancer") OR KW= ("Colorectal Neoplasms") OR KW= ("colorectal cancer") OR KW= ("colorectal Adenoma") OR KW= ("colorectal carcinoma") OR KW= ("colon cancer") OR KW= ("rectal cancer") OR KW= ("rectal Neoplasms")  AND  TI= ("Observational") OR TI= ("Follow up") OR TI= ("Follow-up") OR TI= ("Prospective") OR TI= ("Nested case-control") OR TI= ("Relative risk") OR TI= ("Risk ratio") OR TI= ("Hazard ratio") OR TI= ("odds ratio") OR TI= ("longitudinal") OR TI= ("prospectively") OR TI= ("cohort") OR ("observations") OR TI= ("Cross-Sectional Study") OR TI= ("Case-control Study") OR TI= ("Retrospective Studies") OR TI= ("Retrospective Study") OR AB= ("Observational") OR AB= ("Follow up") OR AB= ("Follow-up") OR AB= ("Prospective") OR AB= ("Nested case-control") OR AB= ("Relative risk") OR AB= ("Risk ratio") OR AB= ("Hazard ratio") OR AB= ("odds ratio") OR AB= ("longitudinal") OR AB= ("prospectively") OR AB= ("cohort") OR ("observations") OR AB = ("Cross-Sectional Study") OR AB= ("Case-control Study") OR AB= ("Retrospective Studies") OR AB= ("Retrospective Study") OR KW= ("Observational") OR KW= ("Follow up") OR KW= ("Follow-up") OR KW= ("Prospective") OR KW= ("Nested case-control") OR KW= ("Relative risk") OR KW= ("Risk ratio") OR KW= ("Hazard ratio") OR KW= ("odds ratio") OR KW= ("longitudinal") OR KW= ("prospectively") OR KW= ("cohort") OR ("observations") OR KW= ("Cross-Sectional Study") OR KW= ("Case-control Study") OR KW= ("Retrospective Studies") OR KW= ("Retrospective Study") |

| **Supplementary Table 2.** Study quality assessment by a 9-point Newcastle-Ottawa Scale for cohort studies. | | | | | | | | | |
| --- | --- | --- | --- | --- | --- | --- | --- | --- | --- |
| Author (Year) | Selection bias | | | | Comparability | Outcome | | | Quality |
|  | Q1 | Q2 | Q3 | Q4 | Q5 | Q1 | Q2 | Q3 |  |
| Basset | * | * | * | * | ** | * | * | * | Good |
| Brink | * | * | * | * | ** | * | - | * | Good |
| Flood | * | * | * | * | * | - | * | * | Good |
| Gibson | * | * | * | * | ** | * | * | * | Good |
| Giovannucci | * | * | * | * | ** | * | - | * | Good |
| Harnack | * | * | * | * | ** | * | * | - | Good |
| Kabat | * | * | * | * | ** | * | * | * | Good |
| Larsson | * | * | * | * | ** | * | * | * | Good |
| Razzak | * | * | * | * | ** | * | * | - | Good |
| Rhonda | * | * | * | * | ** | * | * | * | Good |
| Schernhammer | * | * | * | * | ** | - | * | - | Good |
| Shrubsole | * | * | * | * | ** | * | - | * | Good |
| Su | * | * | * | * | * | * | * | * | Good |
| Terry | * | * | * | * | ** | * | * | * | Good |
| Vogel (2006) | * | * | * | * | ** | * | * | * | Good |
| Vogel (2008) | * | * | * | * | ** | * | * | * | Good |
| Wang | * | * | * | * | ** | - | * | * | Good |
| Zhang | * | * | * | * | ** | - | * | * | Good |
| Zscha¨bitz | * | * | * | * | ** | - | * | * | Good |
| A study can be awarded a maximum of one star for each numbered item within the Selection and Outcome categories. A maximum of two stars can be given for Comparability.  **Selection**  1) Representativeness of the exposed cohort  a) truly representative of the average _______________ (describe) in the community **🟑**  b) somewhat representative of the average ______________ in the community **🟑**  c) selected group of users eg nurses, volunteers  d) no description of the derivation of the cohort  2) Selection of the non exposed cohort  a) drawn from the same community as the exposed cohort **🟑**  b) drawn from a different source  c) no description of the derivation of the non exposed cohort  3) Ascertainment of exposure  a) secure record (eg surgical records) **🟑**  b) structured interview **🟑**  c) written self report  d) no description  4) Demonstration that outcome of interest was not present at start of study  a) yes **🟑**  b) no  **Comparability**  1) Comparability of cohorts on the basis of the design or analysis  a) study controls for _____________ (select the most important factor) **🟑**  b) study controls for any additional factor **🟑** (This criterion could be modified to indicate specific control for a second important factor.)  **Outcome**  1) Assessment of outcome  a) independent blind assessment **🟑**  b) record linkage **🟑**  c) self-report  d) no description  2) Was follow-up long enough for outcomes to occur  a) yes (select an adequate follow up period for outcome of interest) **🟑**  b) no  3) Adequacy of follow up of cohorts  a) complete follow up - all subjects accounted for **🟑**  b) subjects lost to follow up unlikely to introduce bias - small number lost - > ____ % (select an adequate %) follow up, or description provided of those lost) **🟑**  c) follow up rate < ____% (select an adequate %) and no description of those lost  d) no statement | | | | | | | | | |

| **Certainty assessment** | | | | | | |  | **Effect** | **Certainty** |
| --- | --- | --- | --- | --- | --- | --- | --- | --- | --- |
| **№ of studies** | **Study design** | **Risk of bias** | **Inconsistency** | **Indirectness** | **Imprecision** | **Other considerations** | **Case/ participants** | **Relative (95% CI)** |  |
| **Colorectal cancer (dietary folate)** | | | | | | | | | |
| 13 | observational studies | not serious | not serious | not serious | not serious | dose response gradient | 15037/696919 | **RR 0.97** (0.95 to 0.99) | ⨁⨁⨁◯ Moderate |
| **Colorectal cancer (total folate)** | | | | | | | | | |
| 6 | observational studies | not serious | not serious | not serious | not serious | dose response gradient | 8890/512813 | **RR 0.98** (0.97 to 0.99) | ⨁⨁⨁◯ Moderate |
| **Colon cancer** | | | | | | | | | |
| 8 | observational studies | not serious | not serious | not serious | serious^a^ | dose response gradient | 2821/316207 | **RR 0.93** (0.88 to 0.99) | ⨁⨁◯◯ Low |
| **Rectal cancer** | | | | | | | | | |
| 5 | observational studies | not serious | not serious | not serious | serious^b^ | dose response gradient | 996/203907 | **RR 0.98** (0.90 to 1.08) | ⨁⨁◯◯ Low |
| Explanations a. Serious imprecision since the 95% confidence intervals fails to exclude important benefit (RR of <0.90). Downgraded.  b. Serious imprecision since the 95% confidence intervals includes no effect (RR of 1.00), but fails to exclude important benefit (RR of <0.90). Downgraded. | | | | | | | | | |

**Supplementary Table 3**. Meta-evidence judgment quality based on the GRADE.


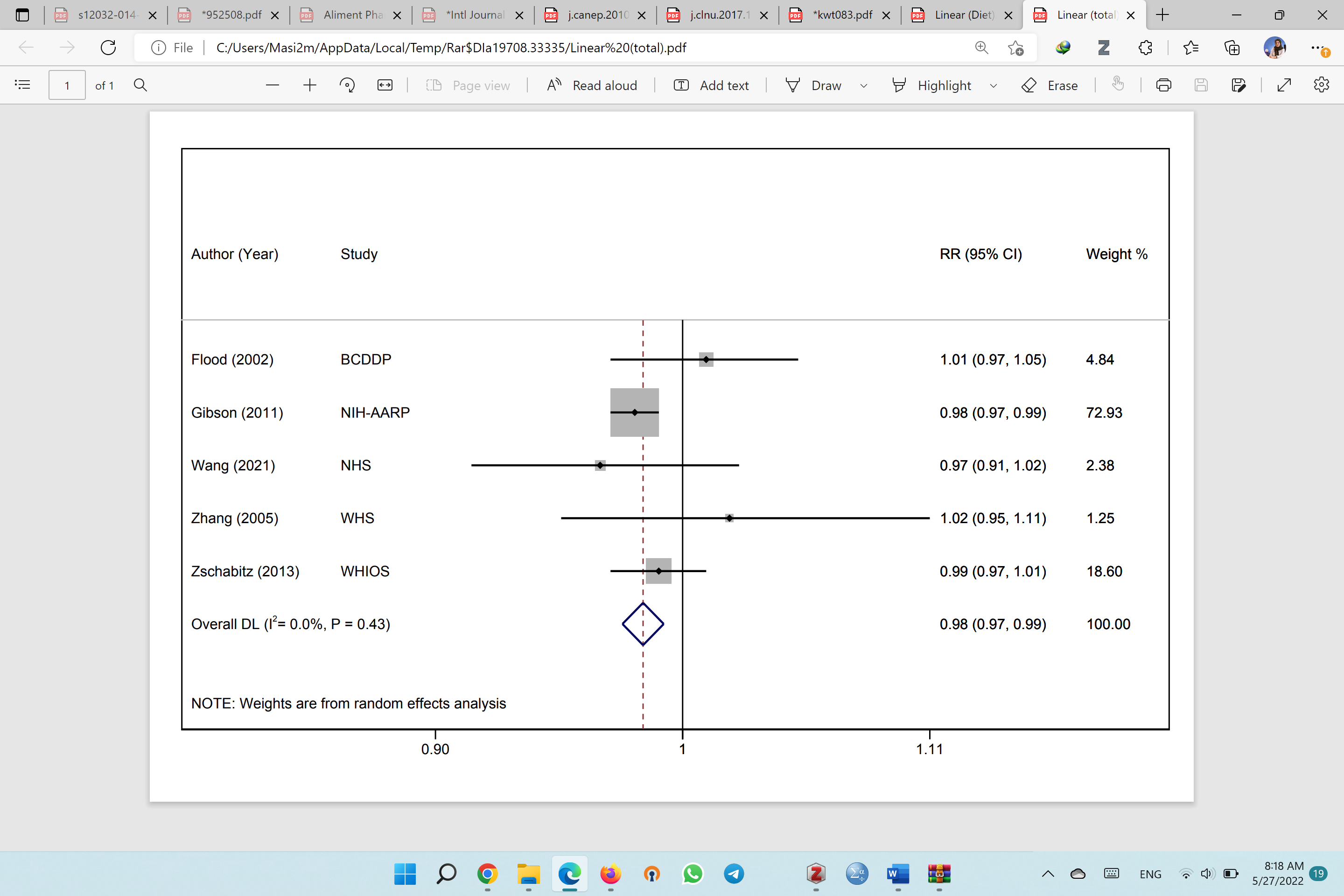


**Supplementary figure 1.** Forest plot for linear dose-response analysis between total folate intake and risk of colorectal cancer.


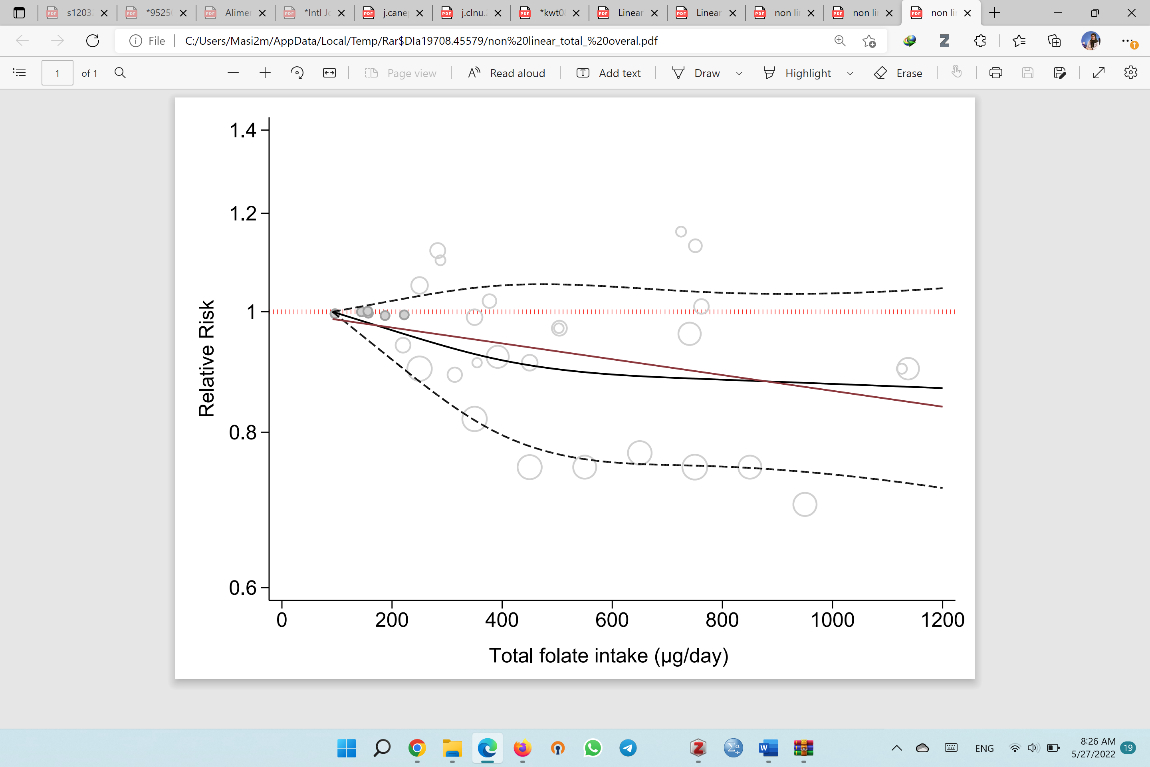

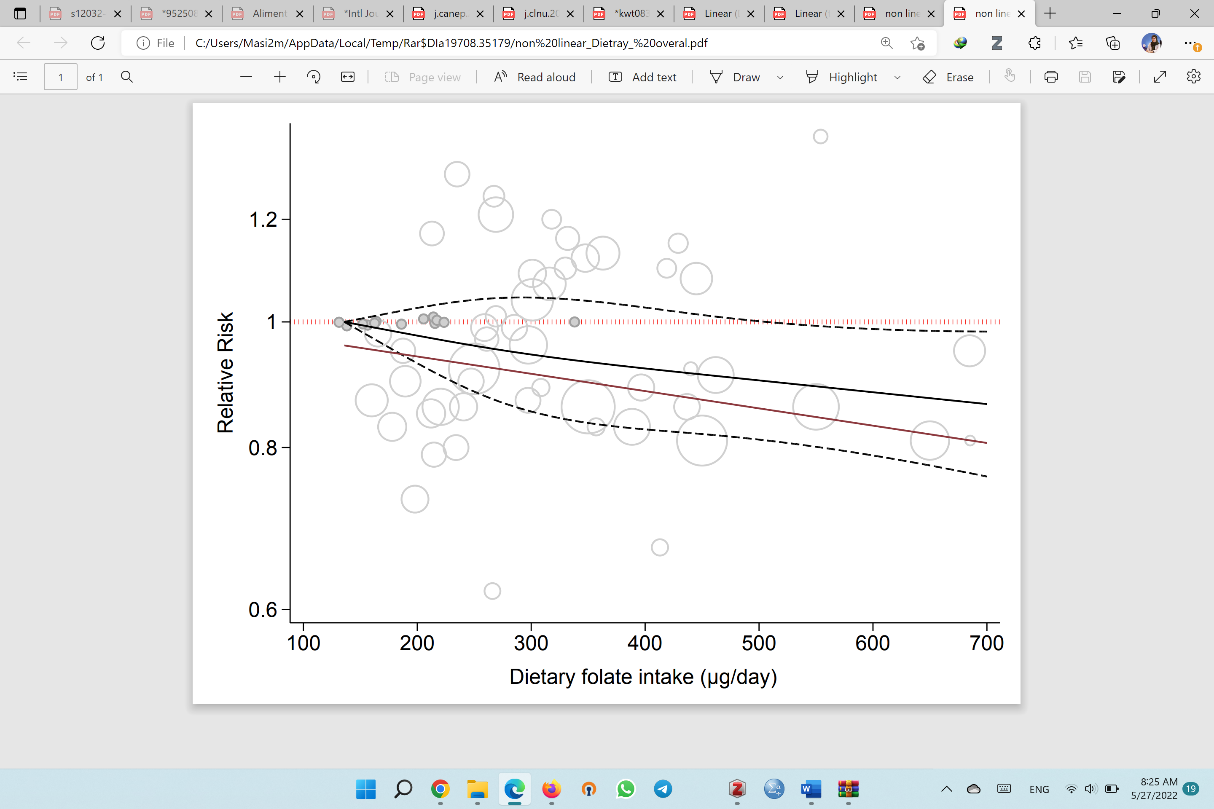


**Supplementary figure 2.** Non-linear dose-response analysis between dietary and total folate intake and risk of colorectal cancer.


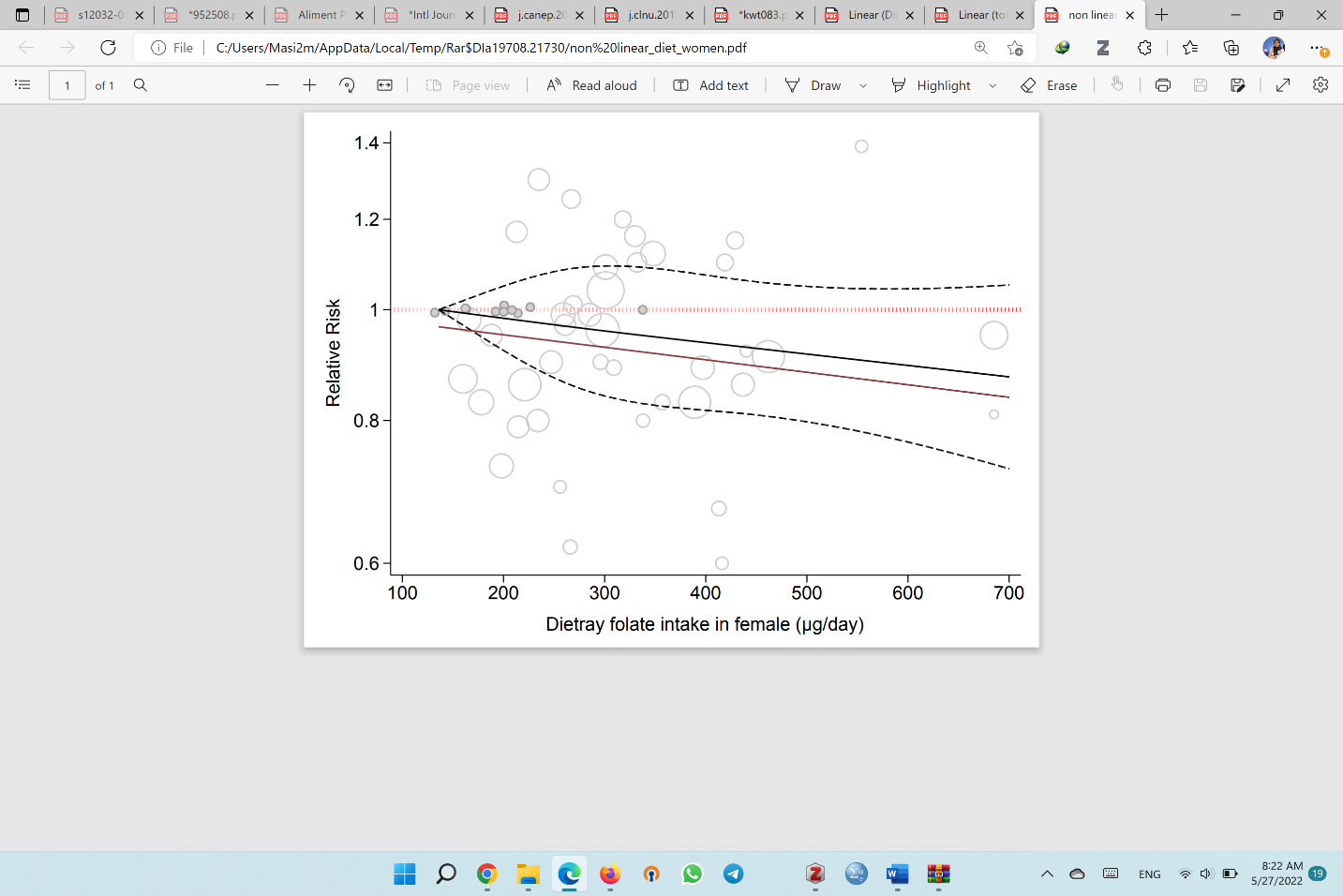

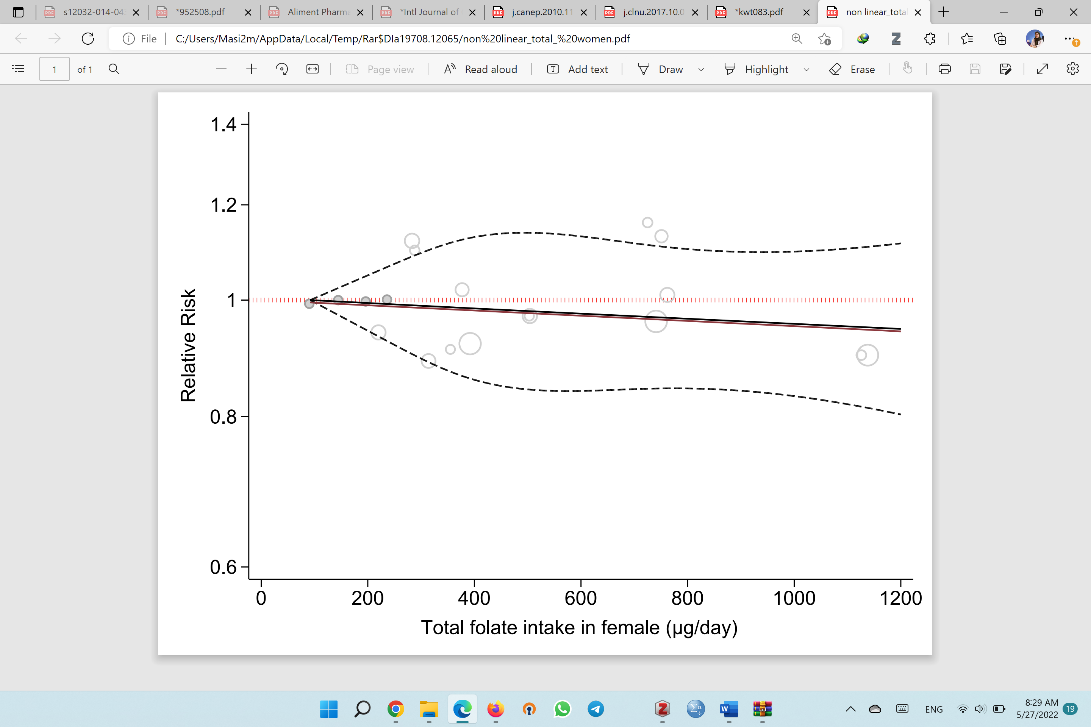


**Supplementary figure 3.** Non-linear dose-response analysis between dietary and total folate intake and risk of colorectal cancer in female.


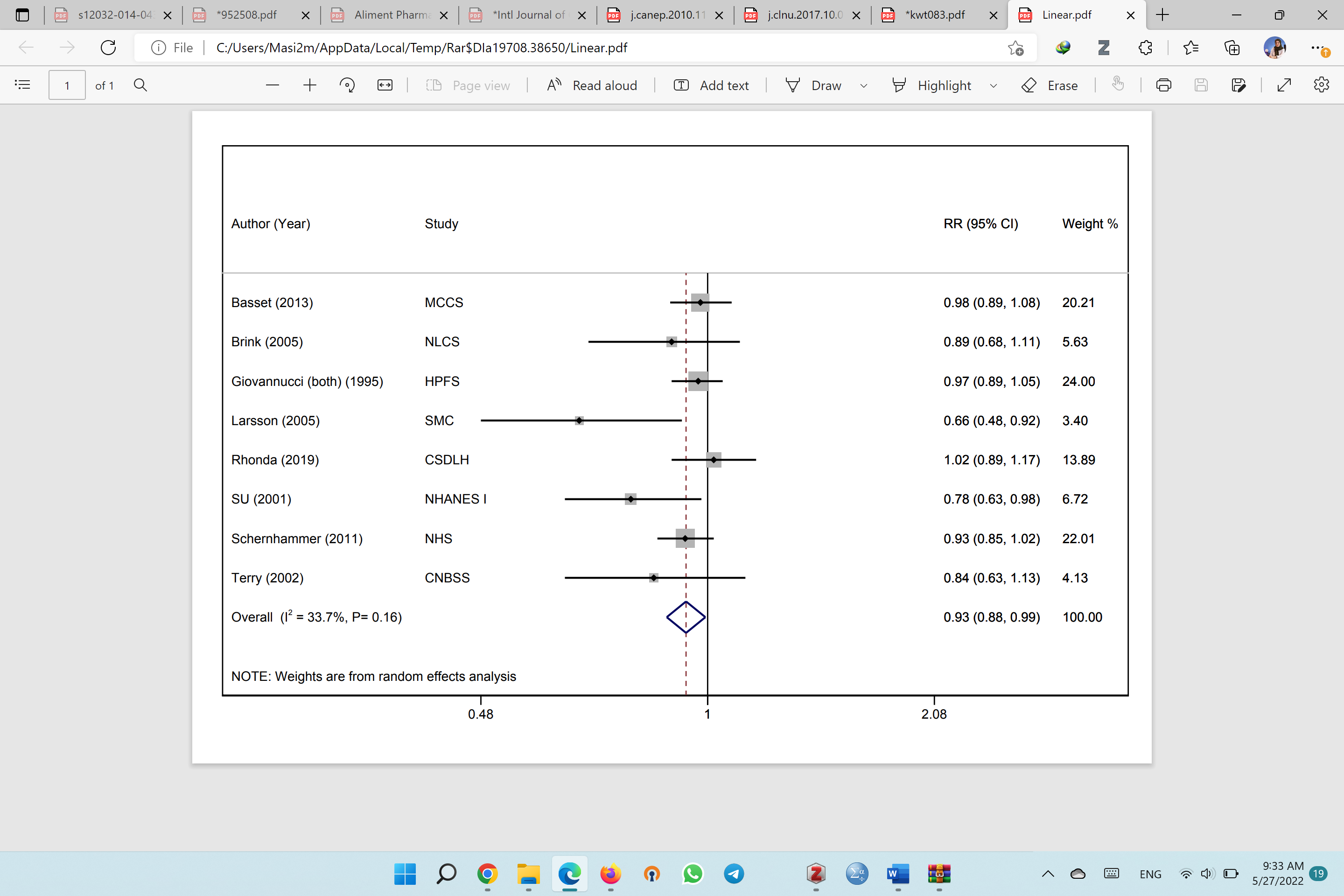


**Supplementary figure 4.** Forest plot for linear dose-response analysis between dietary folate intake and risk of colon cancer.


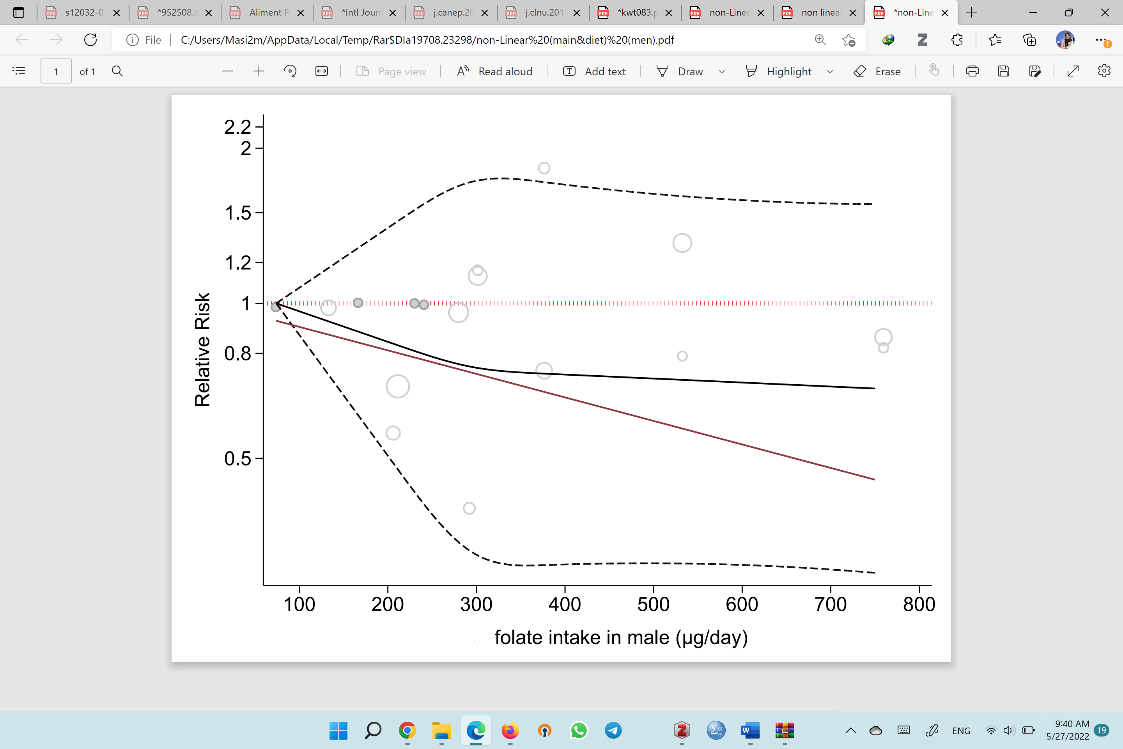

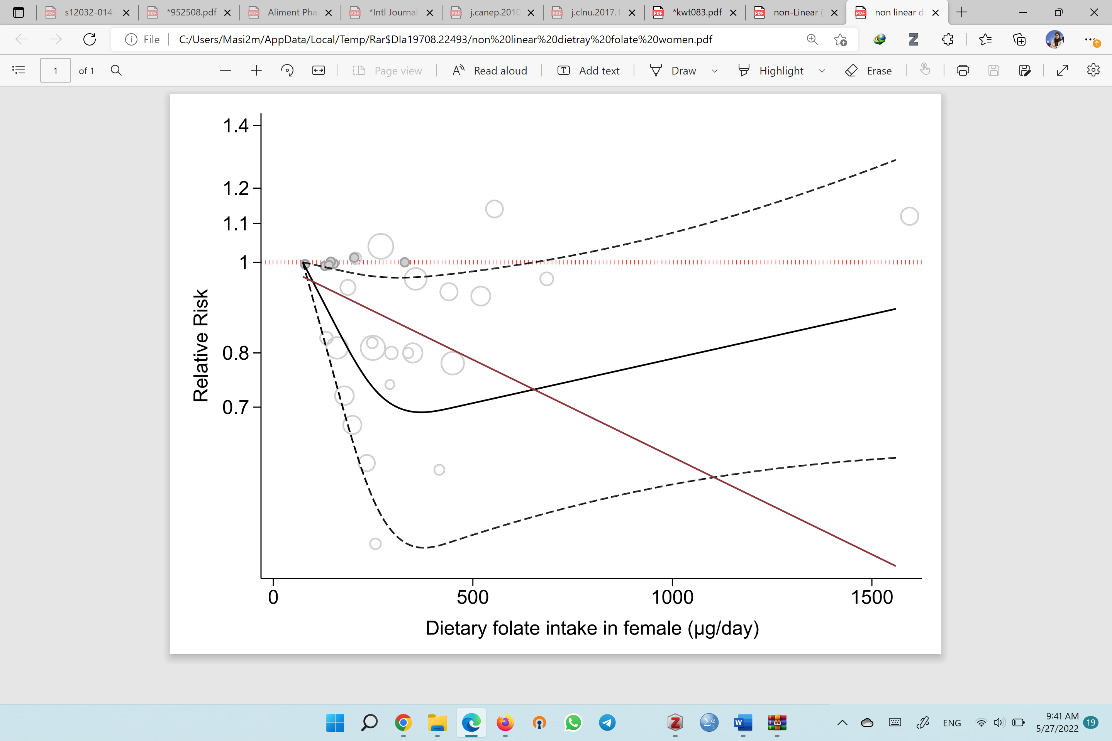


**Supplementary figure 5.** Non-linear dose-response analysis between dietary folate intake and risk of colon cancer in female and male.


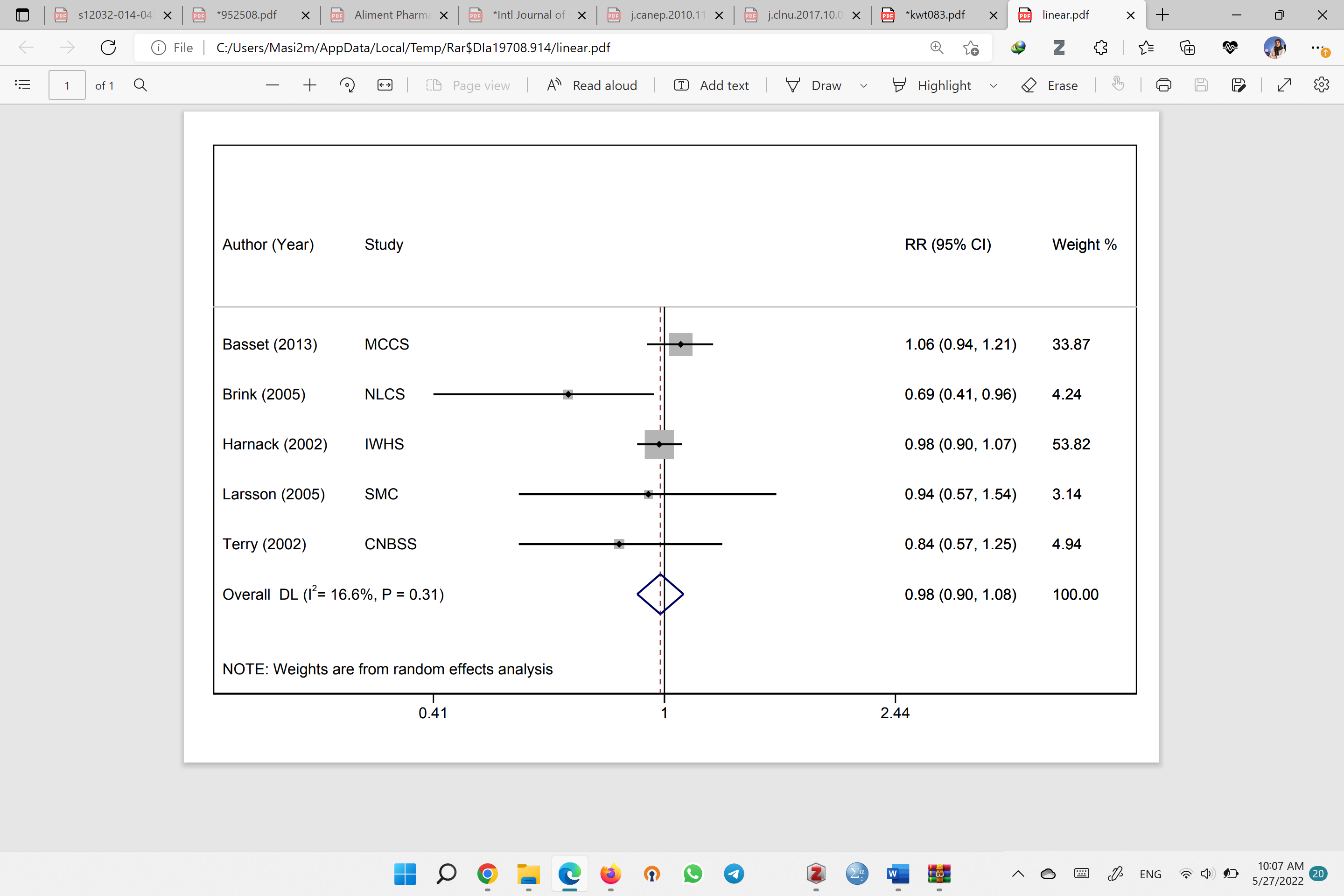


**Supplementary figure 6.** Forest plot for linear dose-response analysis between dietary folate intake and risk of rectal cancer.


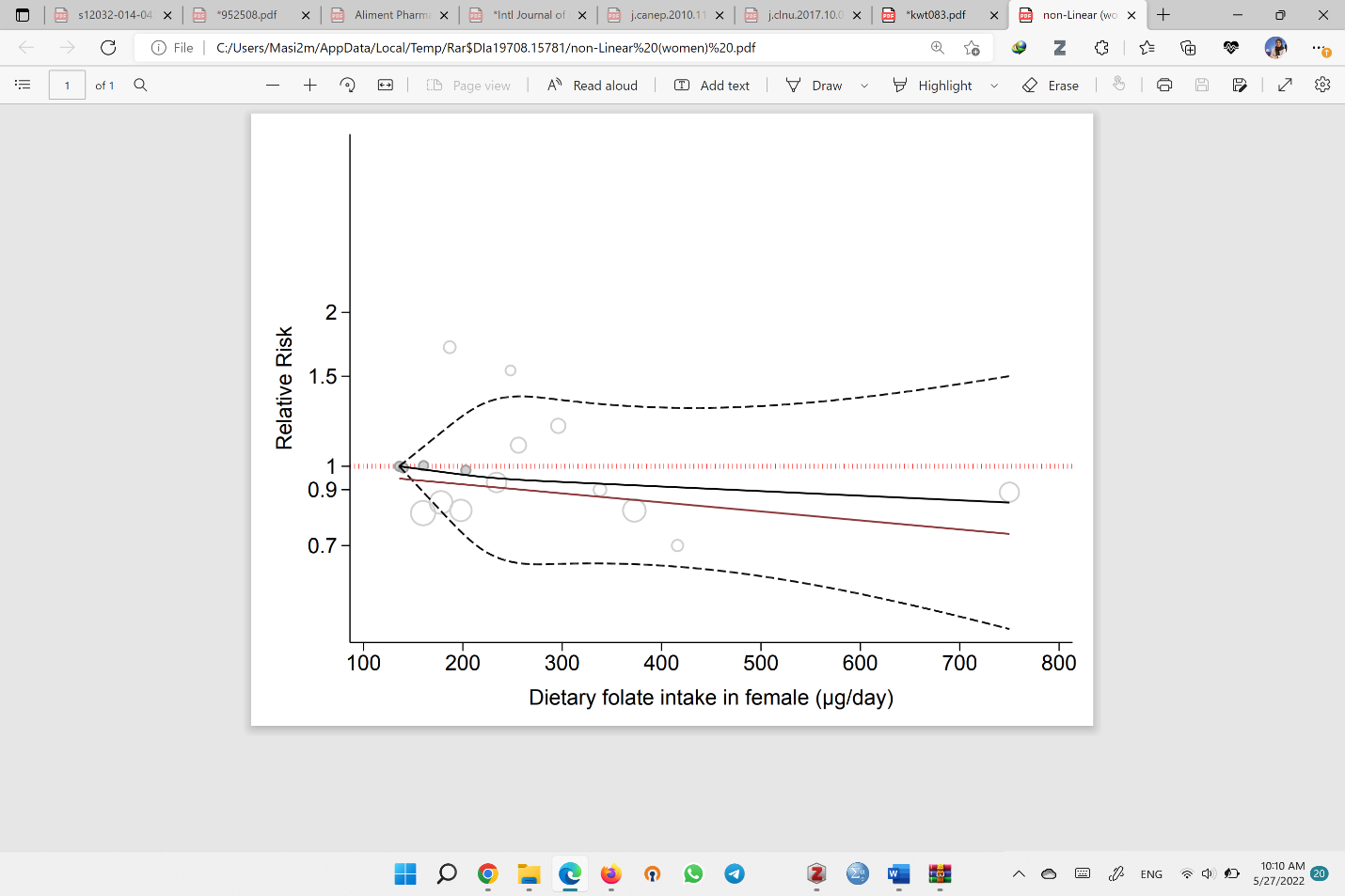

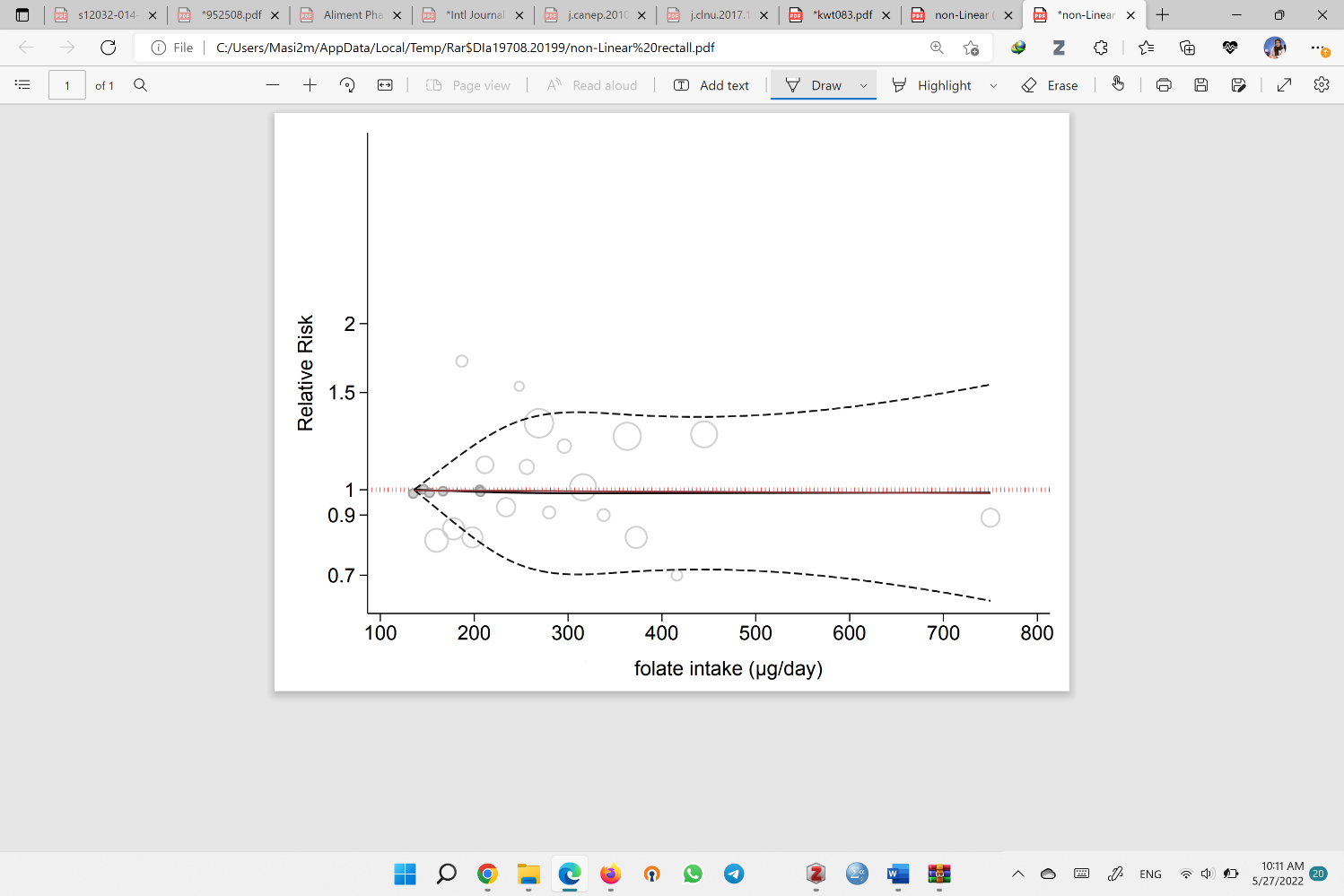


**Supplementary figure 7.** Non-linear dose-response analysis between dietary folate intake and risk of rectal cancer in overall and females.
